# Cross-sensor domain adaptation multi-point monitoring network for mechanical fault diagnosis

**DOI:** 10.1371/journal.pone.0358240

**Published:** 2026-09-11

**Authors:** Zhijie Zhou, Jiao Lu, Yu Ren, Songbin Li

**Affiliations:** 1 School of Electronics & Information Engineering, Wuxi University, Wuxi, China; 2 State Key Laboratory of Rail Transit Vehicle System, Southwest Jiaotong University, Chengdu, China; Zhejiang University, CHINA

## Abstract

In recent years, mechanical fault diagnosis systems based on multi-point sensor data fusion have achieved remarkable advancements. However, they still face several critical challenges: substantial data distribution discrepancies across monitoring points, scarcity of labeled fault samples at newly deployed points, and prohibitive costs of training independent models for each sensor. We propose UCTL, an end-to-end unsupervised cross-sensor transfer learning network that enables the transfer and reuse of fault features from labeled source monitoring points to unlabeled target monitoring points with disparate data distributions, thereby achieving cross-sensor domain adaptation in multi-point monitoring systems. UCTL mainly consists of two major components. First, a one-dimensional (1D) Swin Transformer backbone is developed by modifying the original Swin Transformer. It directly accepts 1D vibration inputs without time-frequency map conversion and efficiently extracts multi-scale and temporally correlated features via shifted-window self-attention. Second, we propose a joint distribution alignment method that extends both Maximum Mean Square Discrepancy (MMSD) and Variance Discrepancy Representation (VDR) to their joint forms (Joint MMSD and Joint VDR). A weighted formulation independently controls the alignment strength of the mean-squared and variance-based distribution discrepancies, improving adaptability across diverse cross-sensor scenarios while mitigating class-mismatch risk. Experiments on six cross-sensor transfer tasks across the CWRU bearing dataset and XJTU Spurgear dataset demonstrate that the proposed method achieves an average diagnostic accuracy exceeding 99%. In addition, initialized with pre-trained parameters from a certain monitoring point, UCTL can significantly reduce the number of training epochs and improve the deployment efficiency of the model. Ablation studies further confirm the superiority of the 1D Swin Transformer backbone and the synergistic effect of the hybrid loss, validating the effectiveness of the proposed approach as a robust cross-sensor domain adaptation approach.

## 1. Introduction

With the growing intelligentization of industrial machinery, the requirements for the accuracy and robustness of mechanical fault diagnosis systems are becoming increasingly stringent. Deep learning techniques have emerged as powerful tools to meet these demands, achieving remarkable success across diverse industrial health monitoring and prognostics tasks. For instance, Zhang et al. proposed a physics-integrated intelligent method that combines wind tunnel experimental data with strip theory simulations to predict propeller aerodynamic properties for electric aircraft, significantly reducing the reliance on costly wind tunnel tests [1]. In the domain of energy storage systems, a self-supervised Informer network was developed for state-of-charge prediction of lithium-ion batteries in electric aircraft, demonstrating superior accuracy under various flight conditions through joint optimization of regression and classification tasks [2]. These advances underscore the broad applicability of deep learning in industrial prognostics and health management. Beyond mechanical systems, deep learning has also driven significant progress in spectral signal processing domains, including hyperspectral target tracking [3] and enhanced Raman spectroscopy [4], demonstrating its versatility across diverse sensing modalities. To comprehensively characterize the operating conditions of industrial facilities, modern fault diagnosis systems often deploy multiple sets of homogeneous sensors (e.g., acceleration sensors) at critical positions, forming a multi-point monitoring network. This design effectively improves the diagnostic accuracy and robustness of the fault diagnosis system [5,6]. However, the increase in the number of sensors also brings new challenges: if an independent diagnostic model is built for each monitoring point, many models need to be trained and maintained separately, leading to waste of computational resources and low efficiency of model updating. Moreover, fault samples of each monitoring point need to be re-collected and re-labeled [7].

In the aforementioned multi-point monitoring scenario, current research primarily focuses on fusing data from multiple sensors. To this end, various multi-sensor data fusion models (MSDF) have been proposed to enhance decision-making accuracy by leveraging information synergy. According to the fusion hierarchy, these can be categorized as follows [8]. Data-level fusion: For example, frequency-domain analysis is used to achieve precise processing of dense raw data, minimizing information loss and realizing the most theoretically accurate fusion [9–11]. Feature-level fusion: This ranges from traditional methods based on time-frequency domain statistical indicators, the wavelet transform, and empirical mode decomposition to deep learning approaches such as Deep Autoencoders (DAE) and Deep Belief Networks (DBN) for extracting and fusing multi-sensor features [12–14]. Decision-level fusion: Voting mechanisms or ensemble methods based on machine learning classifiers are commonly used to integrate the local diagnostic results from each sensor, thereby improving system robustness [15–17].

In addition, in complex working environments, the failure of one or more sensors in multi-sensor monitoring systems often degrades fusion performance or even causes the fusion model to fail. To address this issue, more robust MSDF methods have been proposed. Regarding the construction of sensor fault models, He et al. [18] proposed a Bayesian Convolutional Neural Network (BCNN)-based sensor fusion fault diagnosis framework for high-speed train fault diagnosis systems. This framework accurately distinguishes sensor faults from mechanical faults by merging time-frequency representations based on wavelet, single-sensor spatial features and multi-source temporal features. In terms of adaptive fusion methods, Wang et al. [19] proposed a novel Spatio-Temporal Graph Neural Network with an attention-aware module (A-TSGNN) for multi-sensor fusion fault diagnosis. It models individual sensors using an additional attention module and adjusts each sensor’s importance via flexible weight learning to mitigate the impact of performance degradation in certain sensors during fusion. Regarding redundant complementarity methods for multi-sensor information, Wang et al. [20] proposed an intelligent fault diagnosis model based on mixtures of Gaussians (MoGs) and Variational Autoencoders (VAEs). The model extracts latent variable distributions from multi-sensor data, combines them to form MoGs, and finally obtains fused features via sampling. This method effectively utilizes the redundant complementarity of multi-sensor information, and the model exhibits high robustness against sensor failures and partial signal loss.

The conventional approach to multi-point monitoring is to train an independent diagnostic model for each sensor and fuse them together. This paradigm suffers from three fundamental drawbacks: (i) each new sensor requires re-collection and re-labeling of fault samples, which is costly and sometimes impractical for rare fault modes; (ii) separate models must be trained, stored, and maintained, leading to redundant computational overhead that scales linearly with the number of sensors; and (iii) knowledge learned at one monitoring point cannot be reused at others, resulting in poor deployment efficiency. At their root, all these challenges stem from the same physical reality: fault information travels along distinct propagation paths to each monitoring point, and sensors at different locations exhibit varying intrinsic parameters, collectively resulting in inconsistent data distributions across the monitoring points [21]. By learning domain-invariant features that capture intrinsic fault characteristics rather than sensor-specific variations, a single diagnostic model trained at one monitoring point can be directly transferred to others without target-domain labels. This eliminates the labeling cost at new sensors, enables model reuse across the monitoring network, and allows pre-trained weights to accelerate deployment. In recent years, Transfer Learning (TL) has been increasingly applied to fault diagnosis, with the aim of transferring knowledge learned from source domain data to the target domain to address issues such as target domain data dependency and cross-working-condition challenges [22–25]. Recently, such knowledge transfer mechanisms have been extended to privacy-preserving federated learning frameworks using public data as an intermediary [26]. Essentially, this method relies on a cross-domain feature sharing mechanism between the source and target domains. For multi-sensor monitoring systems, fault features extracted from one monitoring point (defined as source domain) can be transferred to another point (defined as target domain), realizing the reuse and generalization of model capabilities, which is termed “Cross-sensor Domain Adaptation” [27]. Traditional fault diagnosis strategies either train sensor-specific models with heavy annotation costs or aggregate multi-sensor data directly, failing to eliminate inherent distribution shifts among monitoring points [28,29]. In contrast, domain adaptation aligns cross-sensor feature distributions and extracts universal fault-invariant representations, allowing a single well-labeled source-sensor model to generalize to unlabeled target sensors. Compared with conventional signal preprocessing and independent modeling, this method reduces annotation overhead, enables network-wide model reuse, and maintains high diagnostic accuracy under sensor-induced distribution discrepancies. Furthermore, it boasts great compatibility with various backbone networks for flexible deployment. More importantly, it offers a mature, low-cost solution for sensor replacement and new node expansion in multi-point monitoring systems.

Current research in this direction is still in its infancy, but some studies have explored relevant technical paths. Integrating adaptation mechanisms directly into Transformer architectures has emerged as a promising paradigm, such as employing prompt learning to bridge cross-modal distributional gaps [30]. The visual domain adaptation adopted by these methods mainly targets spatial spectrum and illumination features, which differ greatly from the rotational and impact time-frequency characteristics contained in mechanical vibration signals. In terms of mechanical fault diagnosis, several more targeted cross-sensor domain adaptation methods have been proposed. Li et al. proposed a CNN-based domain adaptation method that introduces adversarial training for marginal domain fusion and utilizes unsupervised parallel data to align conditional distributions, thereby addressing machinery fault diagnostics across sensors in different locations [31]. Siahpour et al. proposed a CNN-based cross-sensor domain adaptation method that utilizes Maximum Mean Discrepancy (MMD) and unlabeled parallel data to achieve alignment of the marginal and conditional distributions, respectively, effectively overcoming data distribution discrepancies across sensors [32]. These CNN-based methods are usually constrained by limited local receptive fields and cannot capture global temporal dependencies within vibration signals. To address compatibility issues in heterogeneous sensor data, Transformer architectures and domain adaptation techniques have emerged as research hotspots [30]. Chen et al. proposed an end-to-end cross-sensor domain adaptation method based on an enhanced Transformer and Joint Maximum Mean Discrepancy (JMMD), effectively solving the fault diagnosis problem for electromechanical actuators across different sensor locations [33]. Although this method enhances the model’s extraction capability for local contextual information by loosely coupling convolutional embedding with the basic Transformer, it introduces additional network parameters and computational steps. Moreover, it still consists of multiple structurally identical encoder modules stacked in series, lacking the capability for multi-scale information extraction. In contrast, architectures that can inherently fuse multi-level features have demonstrated superior robustness in challenging scenarios such as motion blur [34], motivating us to explore a backbone capable of comprehensive feature representation. To address the degradation of diagnostic performance under unknown operating conditions, Qin et al. proposed a cross-domain differential attention network (CDDAN) that employs a wavelet principal component feature extraction strategy and a hierarchical differential attention network (HDAN) to extract stable, domain-invariant features [35]. However, CDDAN relies on learned attention weights whose generalization to multi-sensor scenarios with severe distribution shifts remains underexplored. In addition, distribution alignment methods are core approaches for realizing cross-sensor domain adaptation, among which Maximum Mean Discrepancy (MMD) is the most widely adopted metric. However, conventional MMD performs domain alignment merely by aligning the first-order statistic (mean) of data distributions while completely ignoring discrepancies in high-order statistics such as variance, making it difficult to fully represent the cross-domain discrepancy of mechanical vibration signals collected from different monitoring points. To break through the limitations of traditional statistical alignment, Dai et al. proposed a digital twin-assisted graph contrastive domain adaptation (GCDA) method for small-sample bearing fault diagnosis [36]. By aligning data within a graph topological space, this method implicitly captures the high-order structural discrepancies and distributional differences that conventional MMD fails to address, offering a robust paradigm for cross-domain feature mapping under complex operating conditions. However, GCDA requires constructing a faithful digital twin of the monitored equipment, which is not always available in practice.

Given the limitations of the aforementioned methods, the main contributions of this work are summarized as follows:

1. This study addresses a core challenge in industrial multi-point monitoring systems: data distribution discrepancies across sensor positions that hinder cross-sensor knowledge reuse. We propose an end-to-end Unsupervised Cross-Sensor Transfer Learning Network (UCTL), which is developed to address this challenge, enabling fast adaptation of a diagnostic model trained at one monitoring point to others. By initializing with pre-trained model weights of the source monitoring point, it significantly reduces retraining overhead, and alleviates labeled data dependency at target points.
2. A one-dimensional Swin Transformer (1D Swin Transformer) architecture is proposed for the cross-sensor domain adaptation. Through the mechanism of shifted window self-attention, it efficiently captures shared multi-scale fault features and long-range temporal dependencies of vibration signals across various monitoring points. Furthermore, directly operating on raw 1D signals avoids cumbersome preprocessing required by 2D time-frequency transformation, laying a solid foundation for end-to-end cross-sensor transfer learning.
3. An enhanced joint distribution alignment method is proposed in this paper. It extends Maximum Mean Square Discrepancy (MMSD) and Variance Discrepancy Representation (VDR) to their joint forms (JMMSD and JVDR), which are fused through independent weight factors into Joint VDR-MMSD (JVM). The method simultaneously aligns marginal and class-conditional distributions, matches distribution discrepancies across different sensor monitoring points from both mean and variance perspectives, and flexibly balances the mean-squared and variance sensitivity via weight factors, enabling core technical support for accurate cross-sensor domain adaptation.

## 2. Methodologies

### 2.1 Problem definition

This research addresses the challenging problem of cross-sensor transfer learning in mechanical fault diagnosis under multi-point monitoring scenarios. Formally, this task is defined as follows: given a fault diagnosis model trained on data from a specific monitoring point (sensor), our goal is to adapt this model to other monitoring points with different signal distributions. Due to variations in sensor installation positions, signal transmission paths, and operating conditions between different monitoring points, this problem is essentially a domain adaptation task from the source domain (original monitoring point) to the target domain (new monitoring point).

For clarity of problem formulation, the following definitions are given:


$$\begin{array}{c} \begin{array}{cc} & {𝒟}_{s}=\left\{{\left(x_{s}^{i},y_{s}^{i}\right)}_{i=1}^{m},p\right\},\\ & {𝒟}_{t}=\left\{{\left(x_{t}^{i}\right)}_{i=1}^{n},q\right\} \end{array} \end{array}$$ (1)

where *p* and *q* denote the distributions of the source($x_{s}$) and target($x_{t}$) domain samples, respectively. Additionally, $y_{s}$ represents the labels of the source domain samples.

### 2.2 UCTL: Unsupervised cross-sensor transfer learning network

**Fig 1:**
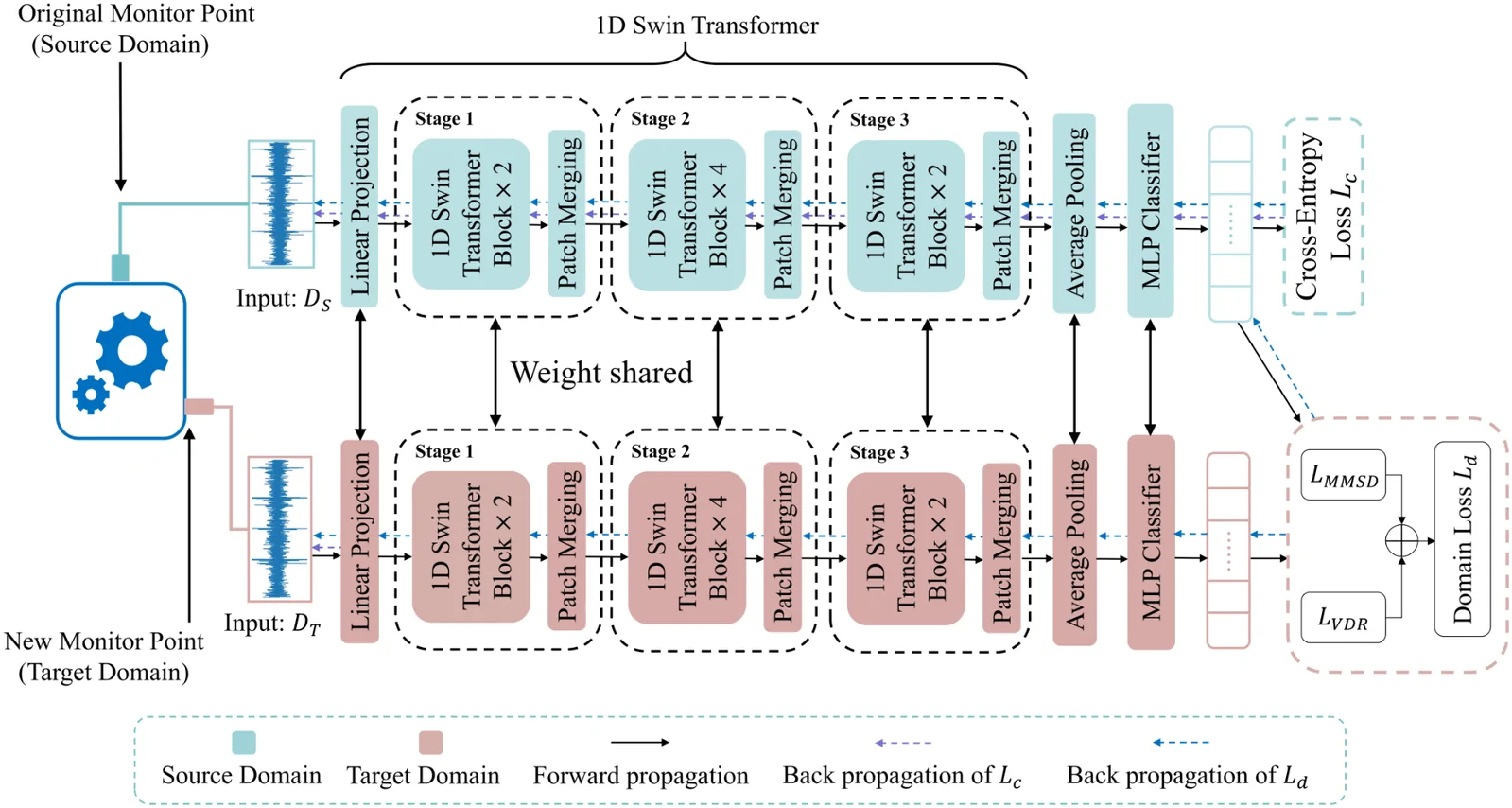
Illustration of the proposed method.


Fig 1 illustrates the proposed cross-sensor domain adaptation framework. The network employs a 1D Swin Transformer as the backbone to extract features from input signals, and the working principles of the backbone are detailed in Section 2.3. During the training phase, both source domain data (${𝒟}_{s}$) and target domain data (${𝒟}_{t}$) are fed into the network. After processing by the shared backbone, feature vectors corresponding to the source and target domains are obtained. During forward propagation, the source and target features are processed independently while sharing all network parameters. During backpropagation, the total loss is a weighted sum of the cross-entropy loss and the domain discrepancy loss, defined as

$$\begin{array}{c} L_{\text{total}}=w_{c}\cdot L_{c}+w_{d}\cdot L_{d} \end{array}$$ (2)

where $L_{c}$ denotes the classification cross-entropy loss, which is calculated only on the labeled source domain data using the extracted features and their ground-truth labels. $L_{d}$ represents the domain alignment loss, which measures the distribution discrepancy between the source and target domain features via the proposed improved distribution alignment method detailed in Section 2.4. $w_{c}$ and $w_{d}$ are the balancing weight coefficients that control the relative importance of the classification loss and the domain alignment loss, respectively.

### 2.3 1D Swin Transformer

The Swin Transformer’s hierarchical shifted window mechanism captures both local details and long-range dependencies [37–40], across all patches in the original image. Its multi-scale architecture enables comprehensive extraction of complex fault features such as periodic impulses and frequency modulation from time-frequency spectrograms [41–44].

Building on these merits, we develop a 1D Swin Transformer (1D-Swin) as the feature extraction backbone, which performs end-to-end learning directly from raw vibration signals without precomputed 2D time-frequency representations. To date, very few studies have deployed a tailored 1D Swin Transformer as the transfer backbone for cross-sensor domain adaptation. Three customizations are made to fit 1D mechanical vibration signals: backbone topology, attention mask calculation after window shifting, and relative position encoding.

Unlike CNNs, which rely on fixed-weight convolutional kernels with inherently limited receptive fields, and conventional Transformers, which adopt global self-attention and single-scale feature modeling that imposes quadratic computational complexity on long vibration sequences, the proposed 1D Swin Transformer incorporates architectural modifications driven by the physical characteristics of mechanical faults. These modifications deliver distinct advantages in both computational efficiency and feature representation for fault diagnosis tasks:

**Linear-complexity processing with global reach**: The global self-attention in conventional Transformers computes pairwise interactions across the entire sequence, yielding *O*(*L*^2^*d*) complexity that becomes prohibitive for high-resolution vibration signals (L≥1024). The 1D Swin Transformer restricts attention to local windows of size *W*, reducing complexity to *O*(*LWd*)—linear in sequence length (detailed in Section 2.3.2) [37]. While CNNs also achieve efficient local computation, their effective receptive fields grow only linearly with network depth, requiring deep stacking to capture long-range temporal dependencies that are critical for distinguishing periodic fault patterns. The shifted window mechanism circumvents this trade-off by enabling cross-window information flow at linear cost.
**Local transient preservation with adaptive attention**: Global attention tends to obscure high-frequency, narrow transients of incipient faults via indiscriminate averaging. Although CNNs can capture local patterns through convolution, their fixed kernel weights apply identical transformations across all signal segments, unable to adaptively emphasize fault-relevant temporal positions. The window-constrained attention in the Swin Transformer provides content-adaptive weighting within local temporal contexts, preserving fine-grained fault signatures [45,46]. Crucially, the shifted window mechanism alternates partition boundaries across adjacent layers, establishing cross-window information pathways that maintain local precision while enabling global context aggregation. This design captures multi-granularity temporal features—from transient impulses to periodic impact cycles—within a unified framework [47,48].
**Construction of hierarchical multi-scale representations and temporal dependencies**: CNNs construct multi-scale feature hierarchies through strided convolution and pooling operations, which irreversibly discard fine-grained temporal information at each downsampling stage. The 1D Swin Transformer adopts a patch merging strategy that concatenates neighboring tokens and linearly projects them into a higher-dimensional space, preserving learnable cross-resolution connections while reducing sequence length. This enables the model to simultaneously maintain high-resolution representations for transient detection and low-resolution abstractions for periodic pattern recognition, which is essential for diagnosing compound fault modes [49,50].

#### 2.3.1 Overall structure.

**Fig 2:**
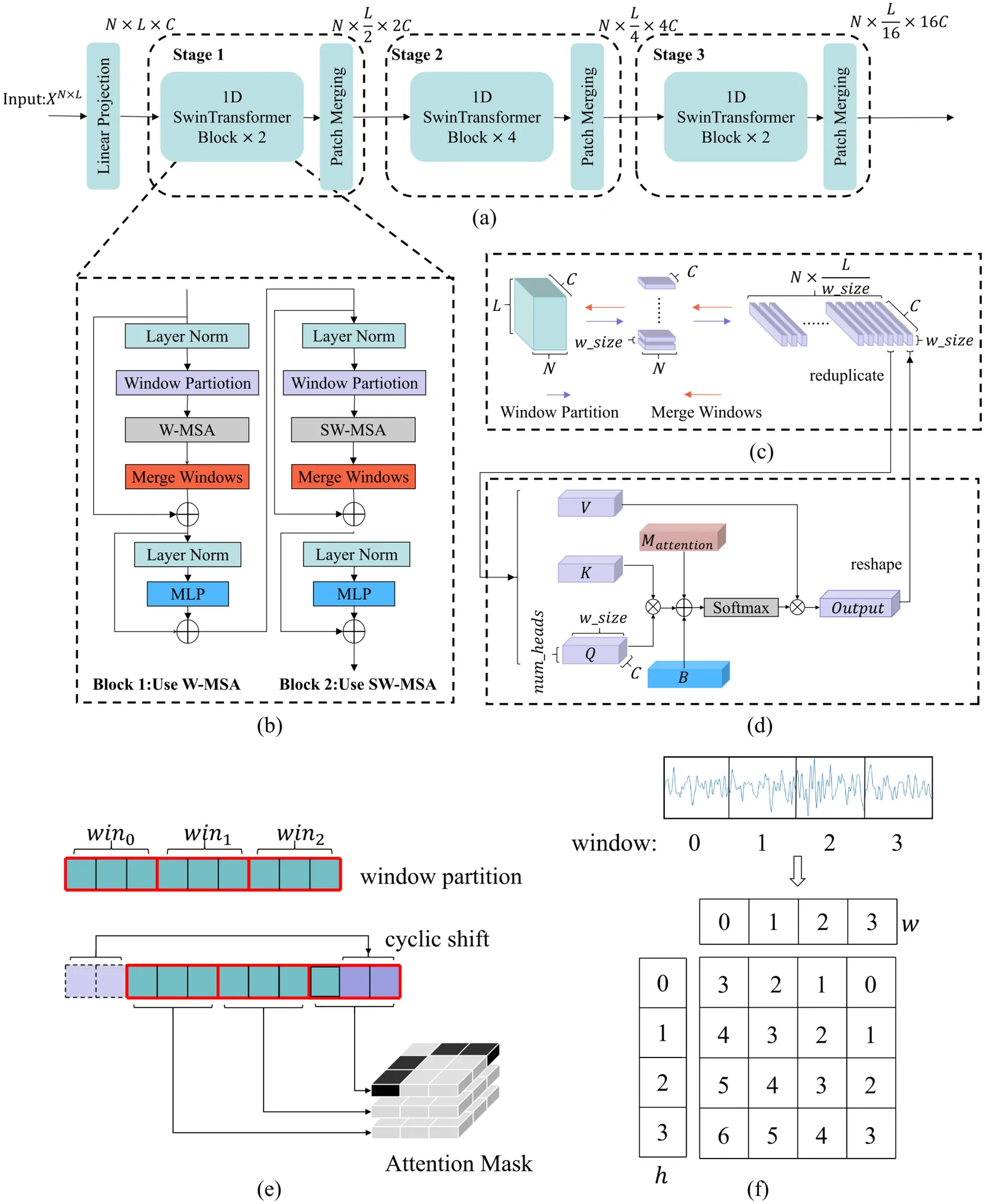
Details of 1D SwinTransformer. (a) The main structure, including 3 stages. (b) Two successive 1D Swin Transformer Blocks. Usually, one block with W-MSA and one block with SW-MSA are connected in series to form a pair. (c) Procedure of Window Partition and Merging for 1D Time Series. It shows the window partition process from left to right and the window merging process from right to left. (d) Computation process of W-MSA and SW-MSA multi-head attention. (e) Shift window mechanism in 1D Swin Transformer Block. It illustrates the shifted window mechanism for 1D time series and depicts the construction of the corresponding attention mask after window shifting. (f) Relative position encoding method in 1D Swin Transformer Block.

Fig 2(a) illustrates the overall architecture of the 1D Swin Transformer. Before being fed into the model, the input signal is split into N non-overlapping segments with identical length L. A linear projection layer maps each segment into a C-dimensional feature vector.

Compared with the original version, the proposed 1D Swin Transformer differs mainly in patch size configuration and stage configuration. First, the patch size is set to 1 instead of the original 4×4. In essence, each physical sampling point of the vibration signal is treated as an individual token. This avoids forced averaging between normal and fault-related samples, enabling effective representation of high-frequency narrow transient impulses induced by incipient faults. Accordingly, the information contained within each token is reduced when the patch size is set to 1, which allows a smaller initial embedding dimension C to mitigate parameter redundancy and moderately balance the overall computational cost.

The token sequence is then processed through three hierarchical stages. Every stage consists of a stack of 1D Swin Transformer Blocks followed by a 1D Patch Merging layer. This layer reduces the length of the feature sequence by half and doubles the channel count, enabling the model to extract multi-scale temporal features at different resolutions.

Compared with the original four-stage 2D Swin Transformer, our three-stage design prevents excessive downsampling to preserve incipient fault features and constrains the upper bound of feature dimension C, which retains useful fault information while suppressing domain-specific noise inherent to individual sensors. In addition, outputs from the three stages correspond to high, medium and low temporal resolutions respectively [37]. Since the middle stage is tasked with modeling periodic correlations and modulation characteristics of fault impulses, it requires more attention blocks for feature extraction [42,51]. Accordingly, computational resources are deliberately allocated to this intermediate-scale module.

#### 2.3.2 1D Swin Transformer block.

Fig 2(b) depicts the internal structure of the proposed 1D Swin Transformer Block, which follows the residual design principle. Each block consists of a layer normalization (LN) layer, a multi-head self-attention module, another LN layer, and a multi-layer perceptron (MLP). To adapt to 1D vibration signals, we modify the 2D window mechanism to its 1D counterpart: before attention computation, the input sequence is divided into non-overlapping windows of fixed size *w*; after attention computation, the window-wise outputs are merged back into a complete sequence. Fig 2(c) provides a schematic illustration of this 1D window partitioning and merging process.

A pair of W-MSA and SW-MSA blocks constitutes a basic building unit, which can be stacked multiple times to extract hierarchical multi-scale features. Fig 2(d) shows the detailed computation flow of multi-head self-attention within a single window, and the corresponding mathematical formula is given by


$$\begin{array}{c} \mathrm{Attention}(Q,K,V)=\mathrm{Softmax}\left(QK^{T}/\sqrt{d}+M_{\text{attention }}+B\right)V \end{array}$$ (3)

where $Q,K,V\in {\mathbb{R}}^{n\times w\times d}$ denote Query, Key, and Value, respectively, with *n* being the number of attention heads, *w* the window size, and *d* the dimension. $M_{attention}$ is the mask of the attention matrix, which is used to address the problem of non-adjacent elements within windows after SW-MSA (to be elaborated in Section 2.3.3). *B* is the relative position encoding matrix shown in Fig 2(f) (to be elaborated in Section 2.3.4).

#### 2.3.3 Mechanism of 1D shifted window and attention mask.

To enable information exchange between adjacent windows, the Swin Transformer adopts a shifted window partitioning mechanism. Fig 2(e) illustrates this process for 1D time series and the corresponding attention mask construction. Specifically, the input sequence is first segmented into non-overlapping local windows of size *w* (red boxes in the figure).

To address the unwanted attention interactions between non-adjacent elements in the original sequence after window shifting, Algorithm 1 details the complete procedure for generating the 1D shifted window attention mask, including the zero-padding step required when the sequence length *L* is not divisible by the window size *w*. The final mask matrix $M_{\text{attention}}\in {\mathbb{R}}^{L\times L}$ is visualized in Fig 2(e).

**Algorithm 1. Calculation of attention mask for shifted window attention mechanism**

**Require:** *L*: sequence length; *w*: window size; *s*: shift size;

**Ensure:** *M*_attention_: attention mask;

1: $L_{p}\leftarrow \lceil L/w\rceil \times w$ ▷ Step 1: Compute pad size *p*

2: $M\leftarrow \text{zeros}(1,L_{p},1)$

3: $p\leftarrow L_{p}-L$

4: **if** *p* = 0 OR *p* + *s* = *w* **then** ▷ Step 2: Determine segmentation regions

5:  $seg_{1}\leftarrow [L_{p}-w,L_{p})$

6:  $seg_{2}\leftarrow [L_{p}-w,L_{p}-s)$

7:  $seg_{3}\leftarrow [L_{p}-s,L_{p})$

8:  $segs\leftarrow (seg_{1},seg_{2},seg_{3})$

9: **else**

10:  section←2×w−L+s

11:  **if** *p* + *s* > *w* **then**

12:   $seg_{1}\leftarrow [L_{p}-section,L_{p})$

13:   $seg_{2}\leftarrow [L_{p}-section,L_{p}-w)$

14:   $seg_{3}\leftarrow [L_{p}-w,L_{p}-s)$

15:   $seg_{4}\leftarrow [L_{p}-s,L_{p})$

16:  **else**

17:   $seg_{1}\leftarrow [L_{p}-w,L_{p})$

18:   $seg_{2}\leftarrow [L_{p}-w,L_{p}-section)$

19:   $seg_{3}\leftarrow [L_{p}-section,L_{p}-s)$

20:   $seg_{4}\leftarrow [L_{p}-s,L_{p})$

21:  **end if**

22:  $segs\leftarrow (seg_{1},seg_{2},seg_{3},seg_{4})$

23: **end if**

24: label←0 ▷ Step 3: Assign region labels

25: **for** each segment *d* in *segs* **do**

26:  M[:,d,:]←label

27:  label←label+1

28: **end for**

29: $M_{\text{windows}}\leftarrow \text{window\_partition}(M,w)$ ▷ Step 4: Partition windows

30: $M_{\text{attention}}\leftarrow M_{\text{windows}}[:,:,\text{None}]-M_{\text{windows}}[:,\text{None},:]$ ▷ Step 5: Compute $M_{attention}$

31: $M_{\text{attention}}\leftarrow \text{masked\_fill}(M_{\text{attention}}\neq 0,-\infty )$

32: $M_{\text{attention}}\leftarrow \text{masked\_fill}(M_{\text{attention}}=0,0)$

**return** *M*_attention_

#### 2.3.4 1D relative position.

The relative position bias mechanism effectively models local positional relationships between tokens. For 1D vibration signals, we adapt this mechanism to the temporal domain as shown in Fig 2(f). The input sequence is first partitioned into non-overlapping local windows of size *w* (taking *w* = 4 as an example in the figure). Since 1D sequences have only a single temporal dimension, we need to construct one-dimensional relative position indices. As defined in Eq (4), the relative position difference between any two tokens in a window ranges from −(w−1) to w−1. We map these differences to non-negative integer indices from 0 to 2w−2, which are then used to index into the learnable relative position bias table of size 2w−1. This design preserves the ability to model temporal order while maintaining computational efficiency.


$$\begin{array}{c} {\text{RelativeIndex}}_{ij}=(h_{i}-w_{j})+(w-1) \end{array}$$ (4)

### 2.4 Joint distribution alignment with weighted VDR-MMSD for cross-sensor domain adaptation

Distribution alignment methods have been widely adopted in cross-domain adaptation to learn domain-invariant representations for cross-sensor fault diagnosis [52–56]. The most representative metric, Maximum Mean Discrepancy (MMD), quantifies distribution difference via mean embeddings in the RKHS [57]. However, MMD relies on first-order mean statistics, making it incapable of representing transient impulses governed by high-order statistics in mechanical faults.

To overcome this limitation, the Maximum Mean Square Discrepancy (MMSD) metric has been proposed specifically for fault diagnosis applications, which leverages mean square statistics to align the distributions of source and target domains in the RKHS [58]. It is defined as


$$\begin{array}{c} {\mathrm{MMSD}}^{2}\left[H⊗H,{𝒟}_{S},{𝒟}_{T}\right]={\left\|{𝔼}_{x\sim p}\left[k(x,\cdot )⊗k(x,\cdot )\right]-{𝔼}_{y\sim q}\left[k(y,\cdot )⊗k(y,\cdot )\right]\right\|}_{H⊗H}^{2} \end{array}$$ (5)

where k(·,·) is the kernel function that maps low-dimensional samples to the RKHS. By utilizing the tensor product k(x,·)⊗k(x,·) of mapped features, MMSD can comprehensively reflect both the mean and variance information of data samples. Furthermore, vibration signals from mechanical equipment are generally symmetric about the origin and approximately Gaussian. In this scenario, the probability density functions (PDFs) of the data across different operating conditions almost completely overlap in terms of mean statistics, resulting in extremely poor performance of MMD. In contrast, MMSD integrates both mean and variance information, resulting in significantly enhanced capability to characterize cross-domain discrepancies in mechanical vibration signals.

Another representative variance-aware distribution alignment method is the Variance Discrepancy Representation (VDR), which explicitly constructs a discrepancy metric by encoding variance information within the RKHS [59]. The formal definition of VDR is presented as


$$\begin{array}{c} {\mathrm{VDR}}^{2}\left[H⊗H,{𝒟}_{S},{𝒟}_{T}\right]={\left\|E_{x\sim p}\tau (x,\cdot )-E_{y\sim q}\tau (y,\cdot )\right\|}_{H⊗H}^{2} \end{array}$$ (6)

where τ(x,·) is an RKHS basis constructed to capture variance information, which is defined as


$$\begin{array}{cc} \tau (x,\cdot ) & \equiv {\left(\kappa (x,\cdot )-{𝔼}_{x\sim p}\kappa (x,\cdot )\right)}^{⊗2}\\ & =\left(\kappa (x,\cdot )-{𝔼}_{x\sim p}\kappa (x,\cdot )\right)⊗\left(\kappa (x,\cdot )-{𝔼}_{x\sim p}\kappa (x,\cdot )\right) \end{array}$$ (7)

where κ(x,·) is the RKHS mapping basis induced by the kernel function, and ${𝔼}_{x\sim p(x)}\kappa (x,\cdot )$ is the kernel mean embedding of the source domain. In this definition, the mean of the kernel mapping is first removed, and then the second-order structure is constructed via a tensor product to match the statistical properties of the variance. The bilinearity property of the tensor product inner product is leveraged as follows:


$$\begin{array}{c} \left\langle \phi_{1}⊗\phi_{2},\phi_{1}^{\prime }⊗\phi_{2}^{\prime }\right\rangle \equiv \left\langle \phi_{1},\phi_{1}^{\prime }\right\rangle \cdot \left\langle \phi_{2},\phi_{2}^{\prime }\right\rangle \end{array}$$ (8)

to simplify high-dimensional tensor computation into combined operations of kernel functions, without requiring explicit construction of high-dimensional features. It can be seen that VDR measures the difference in the mean-subtracted kernel mapping tensor product, i.e., the variance. Similar to MMSD, its design principle is to map samples to a high-dimensional RKHS via kernel functions. The difference is that it relies entirely on variance information to match the characteristic of “more significant variance differences” in vibration signals and specifically designs a Student kernel function to address the long-tailed distribution of mechanical vibration signals, effectively ignoring interference from outlier samples.

The aforementioned methods demonstrate that exploiting mean and variance statistics within the high-dimensional RKHS is critical for an accurate discrepancy metric of vibration signals. However, MMSD and VDR as originally formulated perform only marginal distribution alignment, i.e., they measure the discrepancy between the overall source and target feature distributions without considering class-conditional structures. In cross-sensor fault diagnosis, different fault types may exhibit distinct amplitude variations and transmission-path characteristics at different measurement points; purely marginal alignment therefore risks class mismatch, where features of different fault types are undesirably mixed across domains.

To overcome this limitation, we extend MMSD and VDR to their joint distribution alignment counterparts — termed Joint MMSD and Joint VDR — by simultaneously aligning the marginal distribution and the class-conditional distributions [33,60].

Specifically, unlike the original MMSD and VDR that operate solely on the marginal feature kernel $K_{f}$, our joint variants construct a tensor-product kernel by taking the Hadamard product of the feature kernel $K_{f}$ and the softmax kernel $K_{s}$,


$$\begin{array}{c} K_{\text{joint}}=K_{f}⊙K_{s} \end{array}$$ (9)

thereby aligning the joint distribution *P*(*f*, *s*) rather than the marginal distribution *P*(*f*) alone. Here, $K_{f}$ is the kernel matrix computed on the *d*-dimensional feature representation $f\in {\mathbb{R}}^{d}$, and $K_{s}$ is the kernel matrix computed on the *C*-dimensional softmax output $s=softmax(z)\in {\mathbb{R}}^{C}$, which encodes the classifier’s decision. By incorporating $K_{s}$, the alignment explicitly enforces consistency of the decision boundaries across domains. The joint MMSD and joint VDR are then formulated as


$$\begin{array}{ccc} L_{\text{MMSD}}^{\text{joint}} & = & \text{MMSD}\;\left(K_{f}^{\text{rbf}}⊙K_{s}^{\text{rbf}}\right), \end{array}$$ (10)

$$\begin{array}{ccc} L_{\text{VDR}}^{\text{joint}} & = & \text{VDR}\;\left(K_{f}^{t}⊙K_{s}^{t}\right) \end{array}$$ (11)

where $K^{rbf}$ is a local Gaussian kernel with exponential decay [58]; $K^{t}$ is a heavy-tailed Student-*t* kernel with polynomial decay [59]; the two serve the MMSD and VDR branches, respectively. MMSD(·) and VDR(·) denote the biased forms of *t*he MMSD and VDR domain losses, respectively, as follows,


$$\begin{array}{cc} MMSD(\cdot ) & =\frac{1}{m^{2}}\sum_{i=1}^{m}\sum_{j=1}^{m}k^{2}\;\left(x_{S}^{i},x_{S}^{j}\right)+\frac{1}{n^{2}}\sum_{i=1}^{n}\sum_{j=1}^{n}k^{2}\;\left(x_{T}^{i},x_{T}^{j}\right)-\frac{2}{mn}\sum_{i=1}^{m}\sum_{j=1}^{n}k^{2}\;\left(x_{S}^{i},x_{T}^{j}\right)\\ {}[6pt]VDR(\cdot ) & =\frac{1}{m^{2}}\sum_{i=1}^{m}\sum_{j=1}^{m}{\tilde{k}}^{2}\;\left(x_{S}^{i},x_{S}^{j}\right)+\frac{1}{n^{2}}\sum_{i=1}^{n}\sum_{j=1}^{n}{\tilde{k}}^{2}\;\left(x_{T}^{i},x_{T}^{j}\right)-\frac{2}{mn}\sum_{i=1}^{m}\sum_{j=1}^{n}{\tilde{k}}^{2}\;\left(x_{S}^{i},x_{T}^{j}\right) \end{array}$$ (12)

k(·,·) denotes an element of the joint kernel *K*_joint_, and $\tilde{k}(\cdot ,\cdot )$ denotes an element of the centered joint kernel ${\tilde{K}}_{\text{joint}}$, obtained via double centering:


$$\begin{array}{c} {\tilde{K}}_{XX}={𝐂}_{m}\;K_{XX}\;{𝐂}_{m},\;{\tilde{K}}_{YY}={𝐂}_{n}\;K_{YY}\;{𝐂}_{n},\;{\tilde{K}}_{XY}={𝐂}_{m}\;K_{XY}\;{𝐂}_{n} \end{array}$$ (13)

with ${𝐂}_{m}={𝐈}_{m}-\frac{1}{m}11^{⊤}$ being the centering matrix. The squaring operations *k*^2^ and ${\tilde{k}}^{2}$ are applied element-wise; the centering in VDR removes the first-order mean embedding in the RKHS, thereby focusing the discrepancy on second-order (variance) structure.

Since MMSD captures discrepancy from a mean-squared perspective by directly squaring the kernel matrix, while VDR approaches it from a pure variance perspective by centering the kernel before squaring to isolate variance, we fuse them into Joint VDR-MMSD (JVM) with independent weight factors, enabling flexible balancing between mean-square and variance sensitivities for more comprehensive distribution alignment. It is defined as


$$\begin{array}{c} L_{d}=w_{v}\cdot L_{\text{VDR}}^{\text{joint}}+w_{m}\cdot L_{\text{MMSD}}^{\text{joint}} \end{array}$$ (14)

Given that the joint alignment in JVM relies on the classifier’s softmax outputs *s* as soft pseudo-labels through the softmax kernel $K_{s}$, the quality of $K_{s}$ directly determines the effectiveness of the joint distribution alignment. At the beginning of training, the classifier is randomly initialized, producing near-uniform softmax outputs s≈1/C, which renders $K_{s}$ uninformative and the joint alignment objective unreliable. To address this cold-start problem, we adopt a two-stage warmup strategy. During the first *N*_warmup_ epochs, only marginal MMSD alignment on $K_{f}$ (without $K_{s}$) is applied, allowing the classifier to learn discriminative features and produce reliable softmax outputs. Once the pseudo-labels become trustworthy, the full JVM joint alignment is activated. Formally, the domain loss at epoch *e* is defined as:


$$\begin{array}{c} L_{d}(e)=\left\{ \begin{array}{cc} MMSD\;\left(K_{f}^{rbf}\right) & \text{if }e<N_{\text{warmup}}\\ {}[6pt]w_{v}\cdot L_{\text{VDR}}^{\text{joint}}+w_{m}\cdot L_{\text{MMSD}}^{\text{joint}} & \text{if }e\geq N_{\text{warmup}} \end{array}\right. \end{array}$$ (15)

where *e* denotes the current epoch. This design ensures that the joint kernel $K_{f}⊙K_{s}$ is only constructed when $K_{s}$ carries meaningful class-discriminative information, thereby preventing the alignment from being misled by random pseudo-labels at early stage of training.

## 3. Results and discussion

Two typical mechanical fault diagnosis datasets (bearing and gearbox) are selected to verify the cross-sensor domain adaptation scheme proposed in this study. The datasets and experimental tasks are elaborated in detail below.

### 3.1 Dataset description

(1) CWRU: The CWRU dataset is widely recognized as a standard reference dataset in the field of bearing fault diagnosis. Its experimental platform consists of a drive motor, a torque transducer, a power testing machine, and multiple test bearings. In the experiments, accelerometers were mounted at three locations, namely Supporting Base Plate (BA), Fan End (FE), and Drive End (DE), to collect the corresponding vibration data. The dataset includes four conditions (0, 1, 2 and 3 horsepower). Moreover, it mainly contains faults of the inner race, ball, and outer race. The outer-race faults are further divided into three categories according to their positions relative to the bearing load zone: 3 o’clock (within the load zone), 6 o’clock (perpendicular to the load zone) and 12 o’clock. Consequently, there are five fault conditions in total: inner-race fault, ball fault, outer-race fault at 3 o’clock, outer-race fault at 6 o’clock, and outer-race fault at 12 o’clock [61].
(2) XJTU Spurgear: The XJTU Spurgear dataset is provided by the Institute of Aero-engine, Xi’an Jiaotong University (XJTU). It contains data from 12 accelerometers mounted at different positions on a gearbox, whose spur gear is designed with four severity levels of artificially induced crack damage. Accordingly, five categories of vibration signals were collected, including the normal condition, crack degree 0.2 mm, crack degree 0.6 mm, crack degree 1.0 mm, and crack degree 1.4 mm. Three different speeds are simulated: 900 r/min (15 Hz), 1200 r/min (20 Hz), and variable speeds from 0 to 1200 r/min and then back to 0 [62].

### 3.2 Fault diagnosis tasks for cross-sensor and implementation details

From each dataset, sensor data from three positions (A, B, and C) were selected to perform six transfer diagnosis tasks: A→B, B→C, A→C, B→A, C→B and C→A. Taking the A→B scenario as an example, this notation denotes transferring the model trained on labeled sensor data at position A (the source domain) to position B (the unlabeled target domain), thus completing the cross-sensor transfer learning task from A to B. Table 1 presents the correspondence between the selected positions (A, B, and C) and the actual sensor positions in the datasets. The fault labels and the health conditions are given in Table 2.

**Table 1:** Correspondence of named and actual positions.

Dataset	Named Position	Actual Position
CWRU	A	DE(Drive End)
	B	FE(Fan End)
	C	BA(Base)
XJTU Spurgear	A	Position 1
	B	Position 7
	C	Position 12

**Table 2:** Fault labels and corresponding health conditions.

Dataset	Label	Health Conditions	Sample Size
CWRU	IR	Fault of inner race	39
	Ball	Fault of ball	39
	OR@6	Fault of outer race centered at 6:00	39
	OR@3	Fault of outer race centered at 3:00	39
	OR@12	Fault of outer race centered at 12:00	39
XJTU Spurgear	NC	Normal condition	398
	CD0.2	Crack degree of 0.2 mm	395
	CD0.6	Crack degree of 0.6 mm	453
	CD1.0	Crack degree of 1.0 mm	404
	CD1.4	Crack degree of 1.4 mm	392

### 3.3 Experimental results

**Fig 3:**
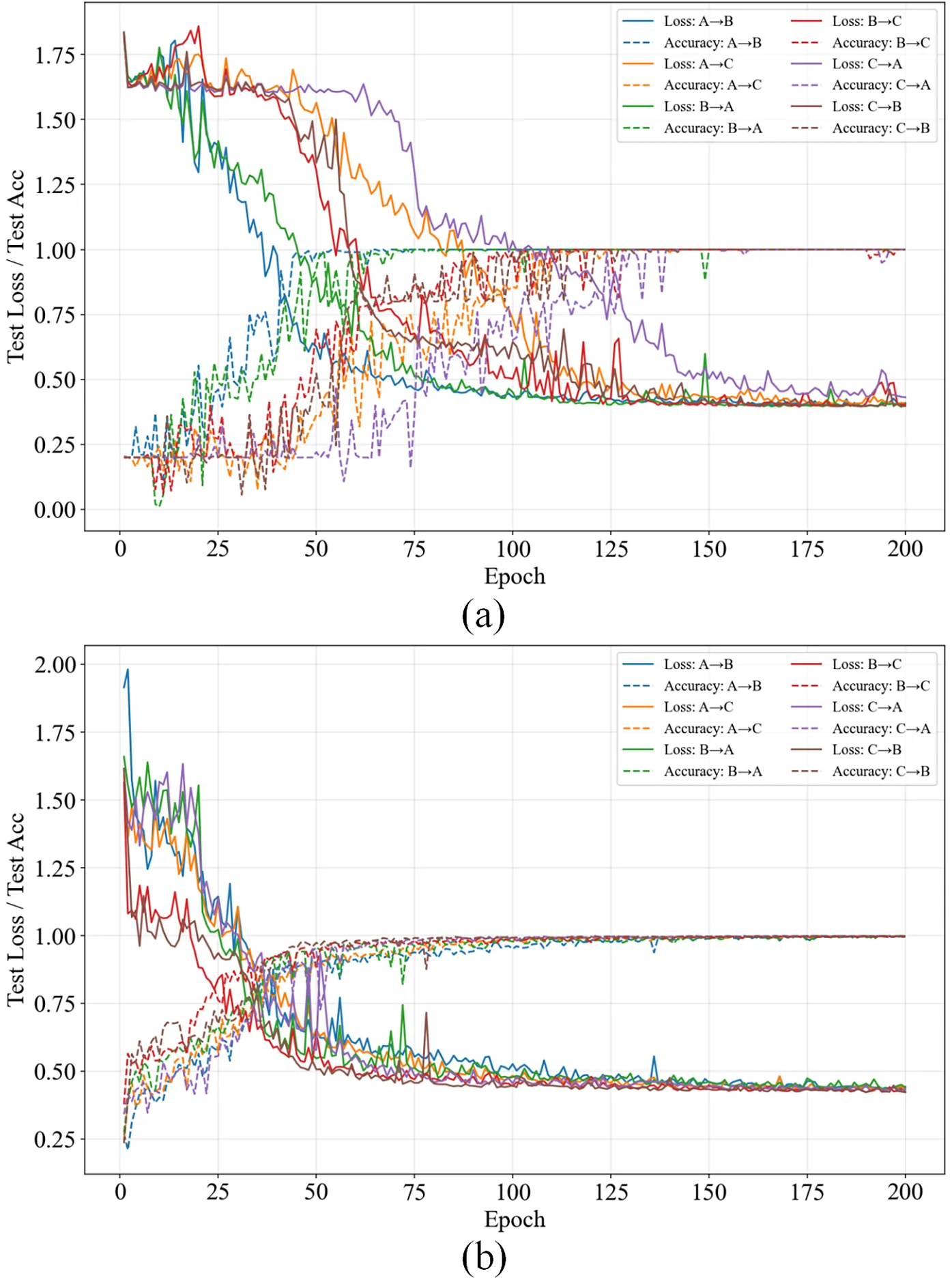
The test losses and accuracies of datasets. (a) Result of CWRU. (b) Result of XJTU Spurgear.

To verify the effectiveness of the proposed UCTL architecture, experiments are conducted on cross-sensor transfer tasks. Fig 3 shows the training curves corresponding to six transfer tasks in the two datasets. As observed, the loss drops rapidly within the first 50 epochs and then decreases gradually before converging steadily to around 0.5. In addition, the training stability on the CWRU dataset is inferior to that on the XJTU Spurgear dataset, which can be mainly attributed to the much fewer available samples for the selected transfer tasks in CWRU. Nevertheless, all tasks eventually achieve convergence, and the test accuracies on both datasets reach nearly 1.0. These results demonstrate the favorable convergence stability of the proposed method.

**Fig 4:**
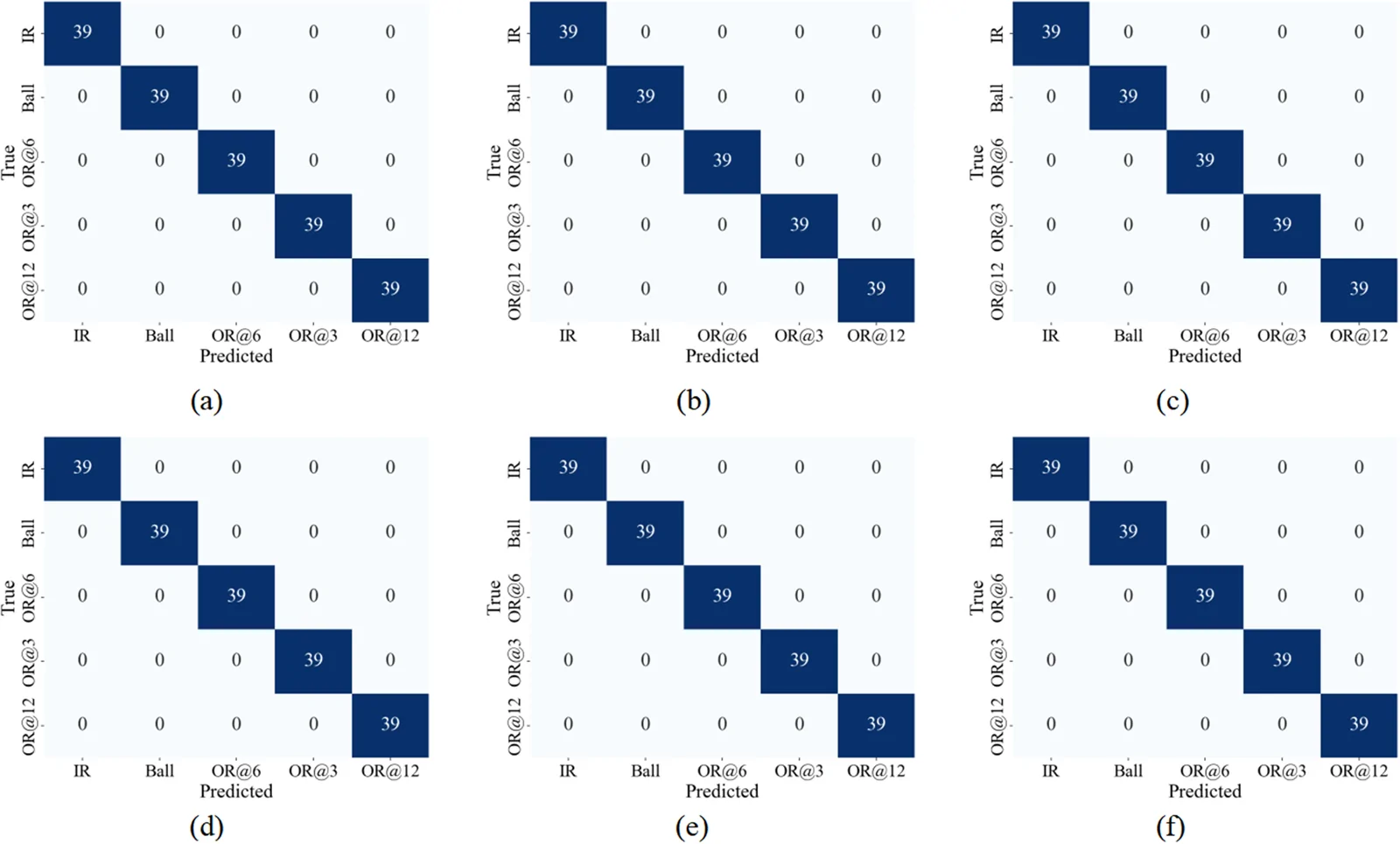
Confusion matrix of cross-sensor tasks in the CWRU dataset. (a) A→B. (b) B→C. (c) A→C. (d) B→A. (e) C→B. (f) C→A.

**Fig 5:**
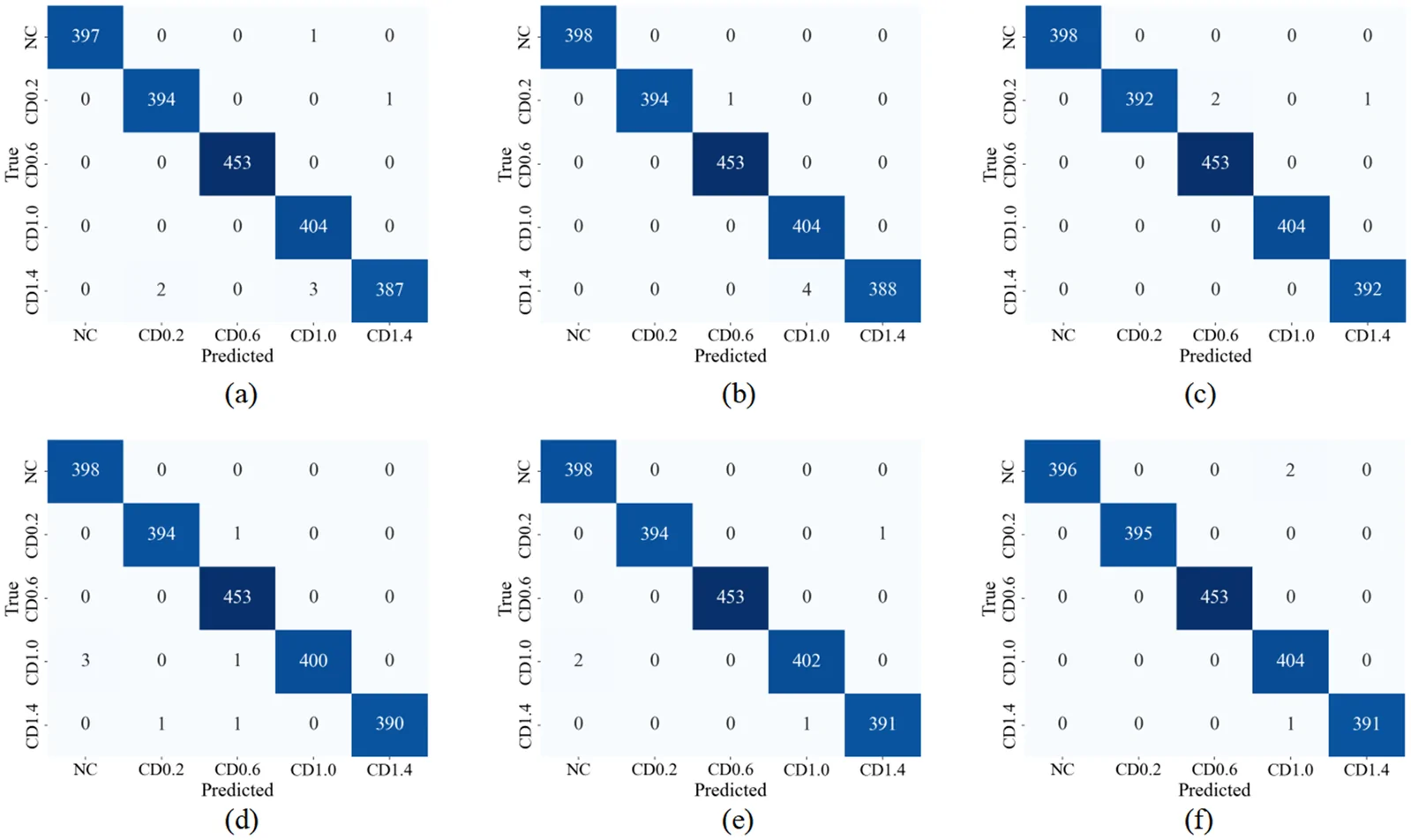
Confusion matrix of cross-sensor tasks in the XJTU Spurgear dataset. (a) A→B. (b) B→C. (c) A→C. (d) B→A. (e) C→B. (f) C→A.

**Fig 6:**
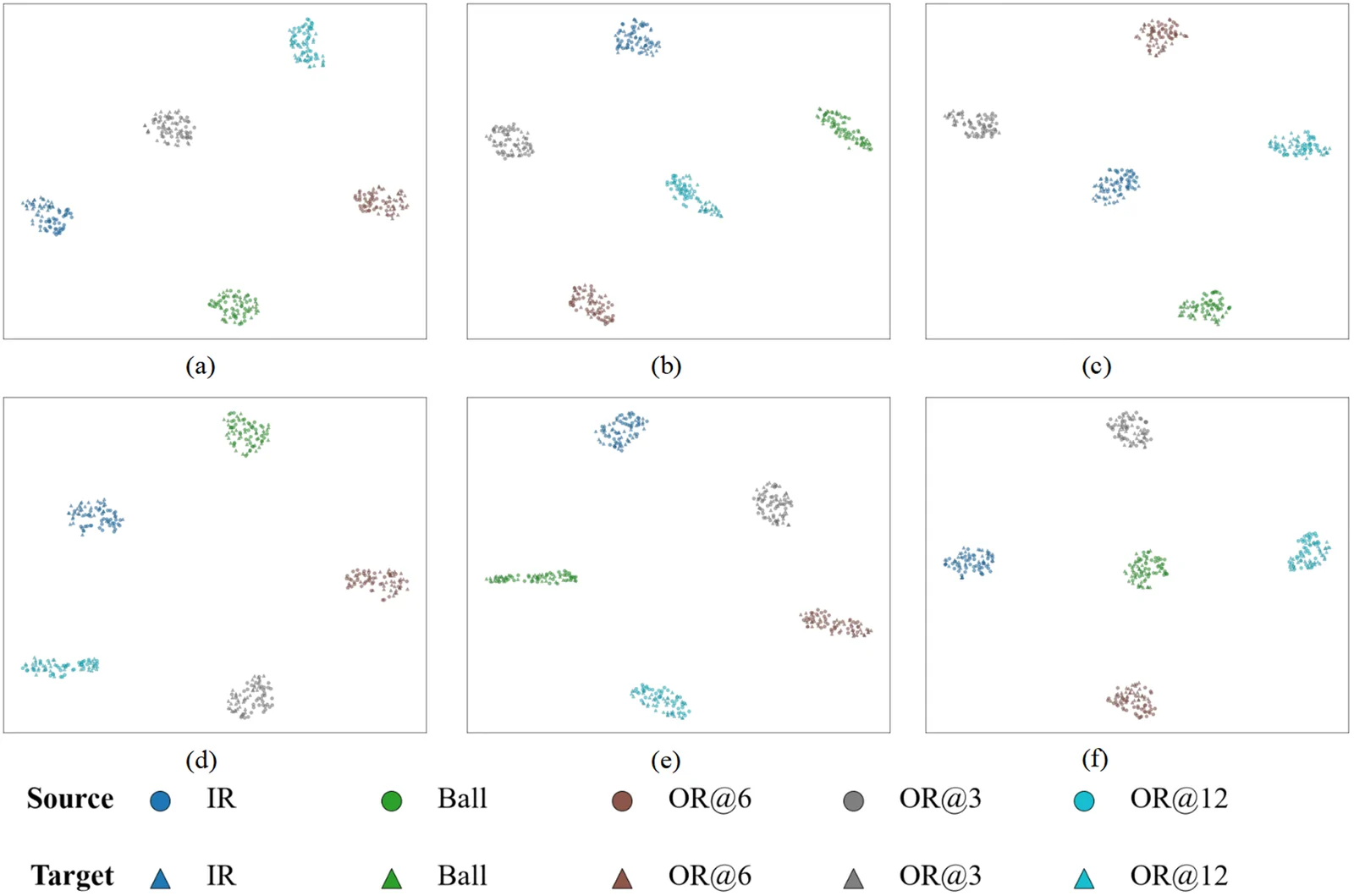
t-SNE of cross-sensor tasks in the CWRU dataset. (a) A→B. (b) B→C. (c) A→C. (d) B→A. (e) C→B. (f) C→A.

**Fig 7:**
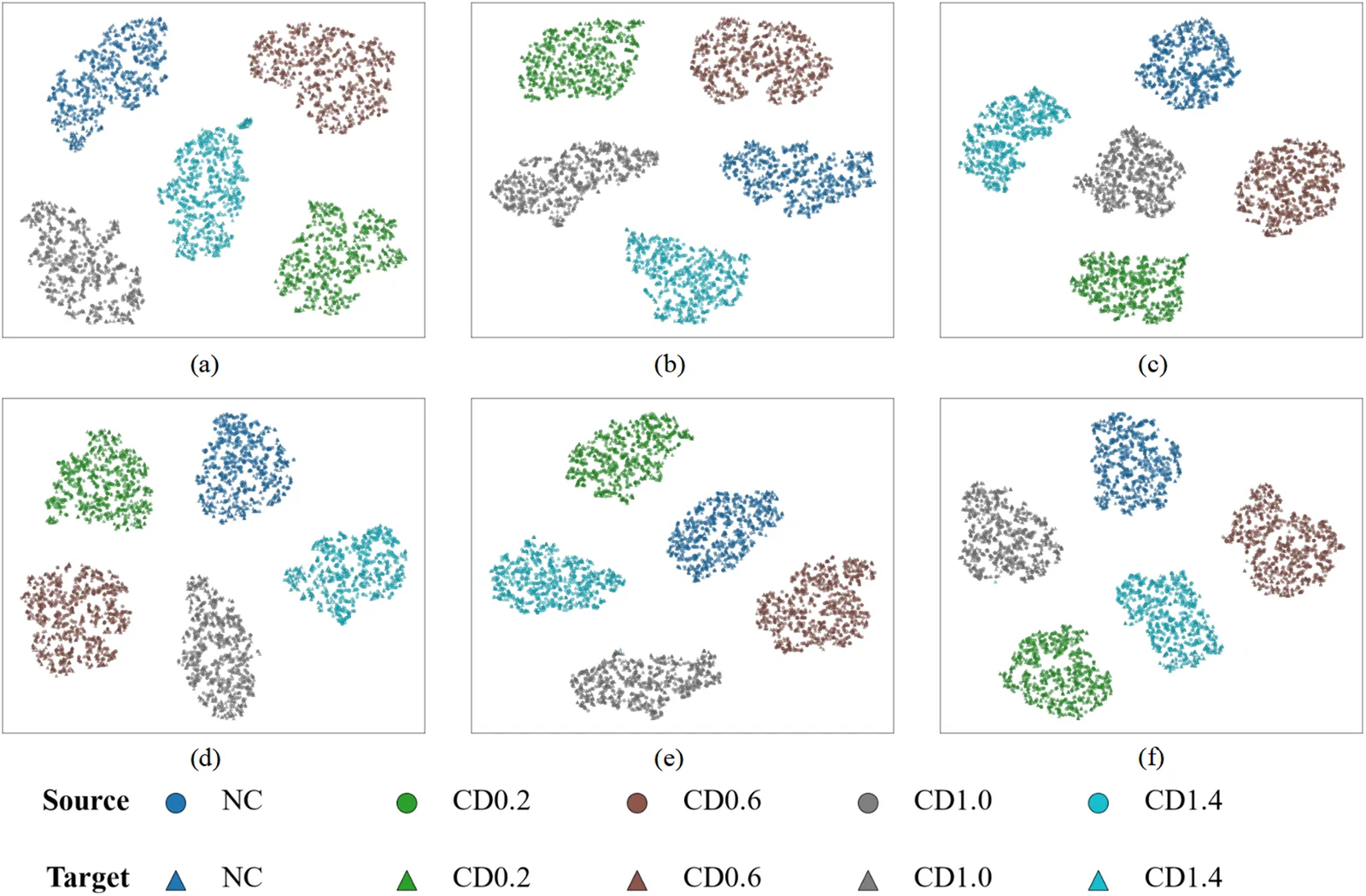
t-SNE of cross-sensor tasks in the XJTU Spurgear dataset. (a) A→B. (b) B→C. (c) A→C. (d) B→A. (e) C→B. (f) C→A.

To comprehensively evaluate the performance of the proposed method, confusion matrices of six transfer tasks on the CWRU and XJTU Spurgear datasets are presented in Figs 4 and 5, respectively. It can be observed that the proposed approach achieves outstanding diagnostic accuracy on the target domain for both datasets: all results exceed 0.99 on the XJTU Spurgear dataset, while all accuracies reach 1.0 on the CWRU dataset. Furthermore, to intuitively visualize the cross-sensor distribution alignment capability of UCTL, t-distributed Stochastic Neighbor Embedding (t-SNE) is adopted to project high-dimensional extracted features into a two-dimensional space, as illustrated in Figs 6 and 7 for the two datasets. Each dot corresponds to an individual sample, where circles denote source-domain samples and triangles denote target-domain samples, and distinct colors represent different health conditions. By employing JVM, which integrates joint distribution alignment (encompassing both marginal and conditional distribution alignment), the same-class samples from the two domains are well aligned. For each health condition, circles and triangles of the same color are tightly clustered together, exhibiting distinct intra-class aggregation and clear inter-class separation. This visualization confirms that UCTL effectively realizes cross-sensor feature distribution alignment, particularly achieving class-conditional alignment between domains.

### 3.4 Training efficiency analysis for newly added monitoring points

To verify that UCTL improves training efficiency when deploying diagnostic models at newly added monitoring points, independent diagnostic models are first trained at position A on both datasets. The learned parameters are then used to initialize the UCTL-based transfer tasks A→B and A→C, respectively.

**Fig 8:**
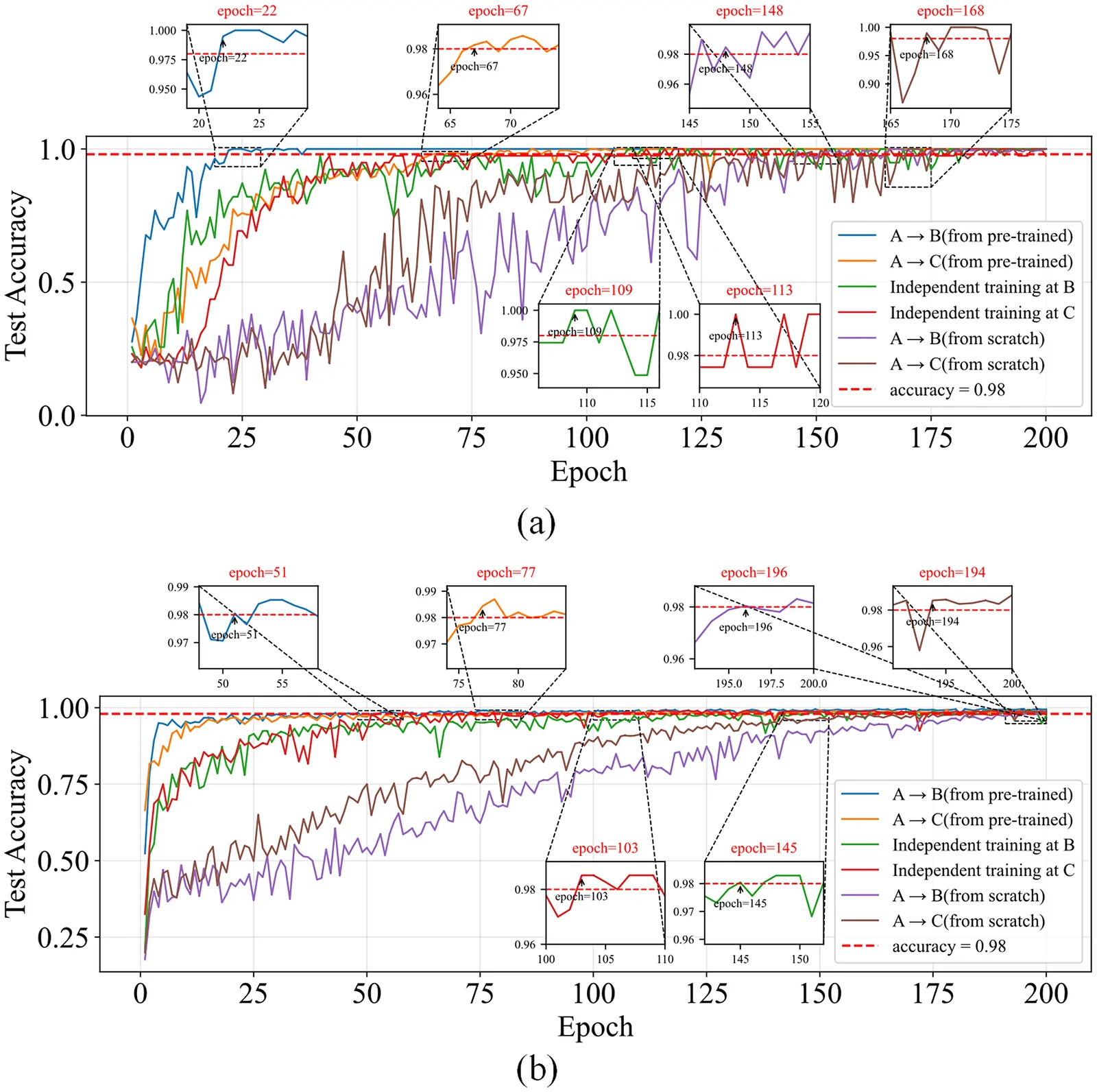
Comparison of model convergence processes under different training strategies. (a) Results of CWRU. (b) Results of XJTU Spurgear.

Fig 8 presents the convergence curves of test accuracy under this strategy, benchmarked against two baselines: independent training directly at positions B and C, and UCTL-based transfer executed from random initialization. A model is considered to have converged when the mean test accuracy over five consecutive epochs first exceeds 98%. For the CWRU, the pretrained-initialization transfers A→B and A→C converge at epoch 22 and epoch 67, respectively. Compared to independent training at positions B and C (109 and 113 epochs), the required epochs are reduced by **79.8%** and **40.7%**, respectively; compared to transfer from random initialization (148 and 168 epochs), the reductions are **85.1%** and **60.1%**. A consistent advantage is observed on the XJTU Spurgear dataset, where pretrained-initialization transfers converge at epoch 51 (A→B) and epoch 77 (A→C). Compared to independent training at positions B and C (103 and 145 epochs), the required epochs are reduced by **50.5%** and **46.9%**; compared to transfer from random initialization (196 and 194 epochs), the reductions reach **74.0%** and **60.3%**. These results demonstrate that initializing with model parameters pre-trained at one monitoring point and subsequently applying UCTL to transfer them to other monitoring points significantly reduces the required epochs, thereby improving the efficiency of model deployment at newly added monitoring points.

### 3.5 Comparative experiment and ablation study

To demonstrate the necessity of integrating the 1D Swin Transformer and the Joint VDR-MMSD fusion module (denoted as JVM) into the proposed UCTL architecture, comparative and ablation experiments were conducted. Specifically, for the tasks described in Section 3.2, we compared the performance of the proposed method against the 1D-SwinT baseline (i.e., the 1D Swin Transformer without distribution alignment). Furthermore, ablation studies were performed on both the backbone network and the distribution alignment module of UCTL, involving five experimental configurations: 1D-SwinT, CNN + JVM, 1D-SwinT + VDR, 1D-SwinT + MMSD and the proposed method (1D-SwinT + JVM). As shown in Table 3, the proposed method achieves test accuracies at or near 1.0 on both datasets, outperforming all other evaluated methods. In contrast, the vanilla 1D-SwinT baseline achieves accuracies below 0.7 on both datasets, thereby validating the effectiveness of the proposed UCTL architecture.

**Table 3:** Test accuracy of the proposed method and other compared methods.

Dataset	Cross sensor	Methods				
		1DSwinT	CNN + JVM	1DSwinT + VDR	1DSwinT+ MMSD	Proposed
CWRU	A→B	0.2666	0.6934	0.3168	0.9867	**1.0000**
	B→C	0.6307	0.5787	0.6307	0.8717	**1.0000**
	A→C	0.2000	0.4266	0.2315	0.9245	**1.0000**
	B→A	0.3230	0.6879	0.2879	0.9784	**1.0000**
	C→B	0.2717	0.6456	0.5692	0.8976	**1.0000**
	C→A	0.2000	0.4109	0.2163	0.9713	**1.0000**
XJTU Spurgear	A→B	0.5279	0.9776	0.4485	0.9784	**0.9966**
	B→C	0.6640	0.9481	0.6958	0.9720	**0.9976**
	A→C	0.6625	0.9497	0.4294	0.8824	**0.9985**
	B→A	0.5357	0.9713	0.4593	0.9284	**0.9966**
	C→B	0.6694	0.9287	0.7105	0.9834	**0.9980**
	C→A	0.4980	0.9426	0.6431	0.9136	**0.9985**

Regarding the ablation of the backbone network, the CNN backbone yields significantly inferior performance on the CWRU dataset compared to the XJTU Spurgear dataset, whereas the 1D Swin Transformer exhibits a negligible performance difference between the two datasets. This is primarily because the CWRU dataset contains far fewer samples under specific operating conditions than the XJTU Spurgear dataset. Traditional CNNs suffer from severe performance degradation in low-data regimes [63,64]. Even when equipped with the JVM distribution alignment module, their feature representation capability remains markedly lower than that of the 1D Swin Transformer. This collectively verifies that the UCTL framework exhibits strong generalization capability and can maintain satisfactory performance on datasets with different sample scales.

In the ablation experiments on distribution alignment method, the vanilla MMSD baseline already achieves a favorable cross-sensor diagnosis accuracy (most tasks yield accuracies over 0.9), as it effectively encodes both mean and variance statistical characteristics of vibration signals, providing a more comprehensive distribution discrepancy characterization than VDR, which only focuses on variance. However, by integrating VDR through a weighted fusion strategy, we further strengthen the model’s ability to capture fine-grained variance differences in vibration signals. This targeted enhancement results in a more pronounced improvement in the final diagnosis performance of UCTL.

To evaluate the impact of key hyperparameters in the 1D Swin Transformer, we further conduct an ablation study on the patch size and stage configuration. The results in Table 4 show that a patch size of 1 with the [2,4,2] stage configuration achieves the best performance, yielding an average accuracy of 1.0000 on CWRU and 0.9976 on XJTU Spurgear. A patch size of 1 preserves the full temporal resolution of vibration signals, enabling fine-grained transient fault features to be retained; larger patch sizes (4, 8) reduce the sequence length and cause up to 4.3% accuracy degradation on XJTU Spurgear. Among stage configurations, [2,4,2] concentrates its representational capacity in the intermediate stage and strikes the best balance between feature abstraction and parameter efficiency, whereas the deeper [2,2,6,2] configuration consistently underperforms due to over-parameterization under limited training samples.

**Table 4:** Ablation of patch size and stage configuration (test accuracy).

Dataset	Task	patch size = 1			patch size = 4			patch size = 8		
		[2,2,2]	[2,4,2]	[2,2,6,2]	[2,2,2]	[2,4,2]	[2,2,6,2]	[2,2,2]	[2,4,2]	[2,2,6,2]
CWRU	A→B	0.9867	**1.0000**	0.9948	**1.0000**	**1.0000**	0.9794	0.9784	0.9743	0.9589
	B→C	0.9948	**1.0000**	0.9897	**1.0000**	**1.0000**	**1.0000**	0.9889	0.9846	0.9743
	A→C	**1.0000**	**1.0000**	**1.0000**	0.9989	**1.0000**	0.9692	0.9981	0.9941	0.9687
	B→A	**1.0000**	**1.0000**	**1.0000**	**1.0000**	**1.0000**	**1.0000**	0.9838	0.9794	0.9692
	C→B	**1.0000**	**1.0000**	0.9897	**1.0000**	**1.0000**	0.9743	0.9730	0.9938	0.9835
	C→A	**1.0000**	**1.0000**	0.9897	0.9941	**1.0000**	0.9794	0.9935	**1.0000**	0.9589
	**Avg**	0.9969	**1.0000**	0.9940	0.9988	**1.0000**	0.9837	0.9860	0.9877	0.9689
XJTU Spurgear	A→B	0.9829	**0.9966**	0.9902	0.9639	0.9924	0.9746	0.9417	0.9702	0.9575
	B→C	**0.9990**	0.9976	0.9917	0.9668	0.9980	0.9775	0.9451	0.9736	0.9609
	A→C	0.9863	0.9985	0.9932	0.9702	**0.9990**	0.9809	0.9485	0.9771	0.9644
	B→A	0.9814	0.9966	**0.9985**	0.9620	0.9810	0.9731	0.9398	0.9683	0.9556
	C→B	0.9853	**0.9980**	0.9912	0.9658	**0.9980**	0.9770	0.9436	0.9721	0.9595
	C→A	0.9834	**0.9985**	0.9907	0.9644	0.9829	0.9751	0.9422	0.9707	0.9580
	**Avg**	0.9864	**0.9976**	0.9926	0.9655	0.9919	0.9764	0.9435	0.9720	0.9593

### 3.6 Parameter sensitivity experiment

To analyze the impact of weight factors on the performance of the proposed method, this study conducted a parameter sensitivity experiment. According to Eq (2), let


$$\begin{array}{cc} & w_{d}=1.0-w_{c},\\ & w_{m}=1.0-w_{v} \end{array}$$ (16)

then we have


$$\begin{array}{cc} & L_{\text{total}}=w_{c}\cdot L_{c}+(1-w_{c})\cdot L_{d},\\ & L_{d}=w_{v}\cdot L_{\text{VDR}}+(1-w_{v})\cdot L_{\text{MMSD}} \end{array}$$ (17)

**Fig 9:**
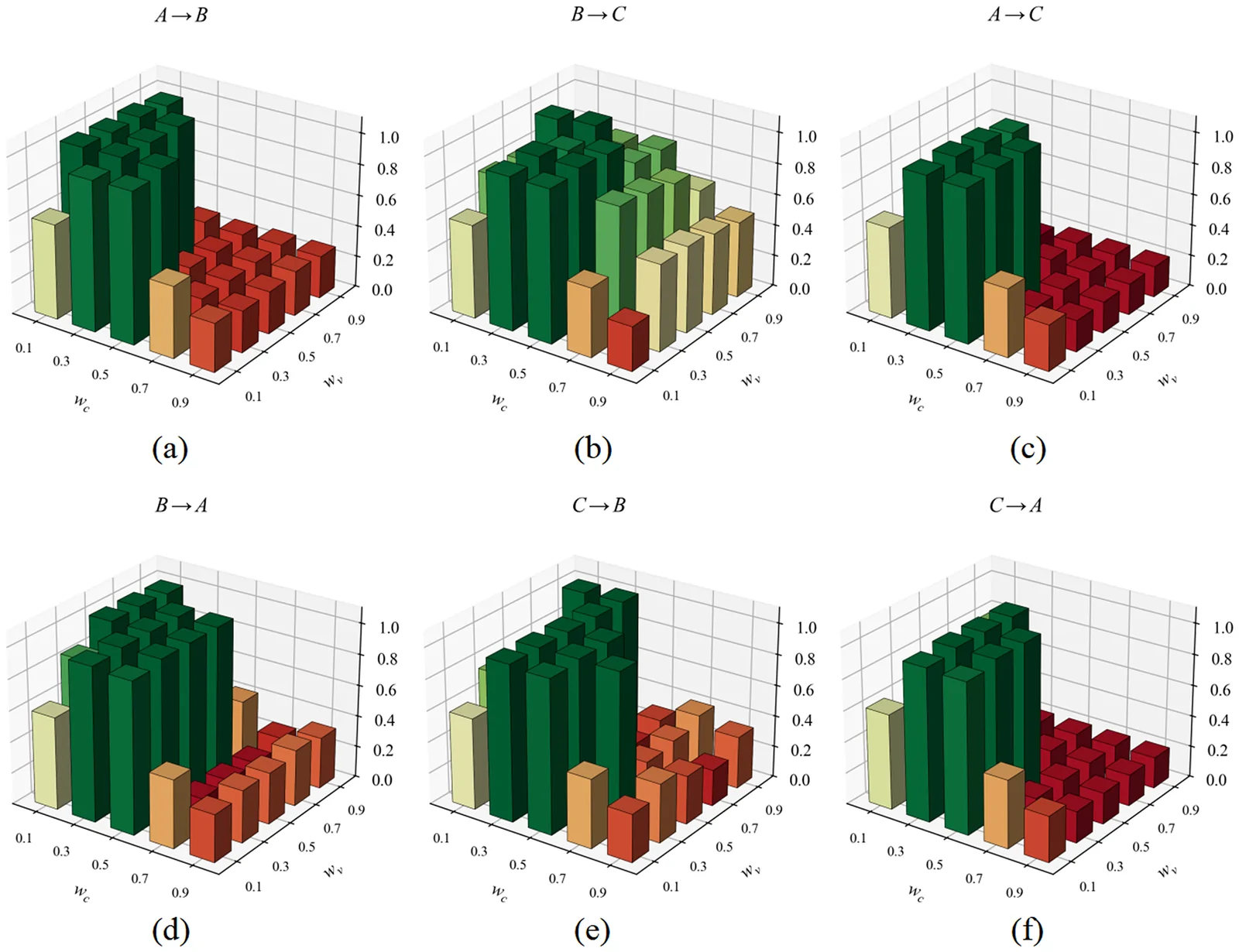
Parameter sensitivity results of $w_{c}$ and $w_{v}$ for CWRU dataset. (a) A→B. (b) B→C. (c) A→C. (d) B→A. (e) C→B. (f) C→A.

**Fig 10:**
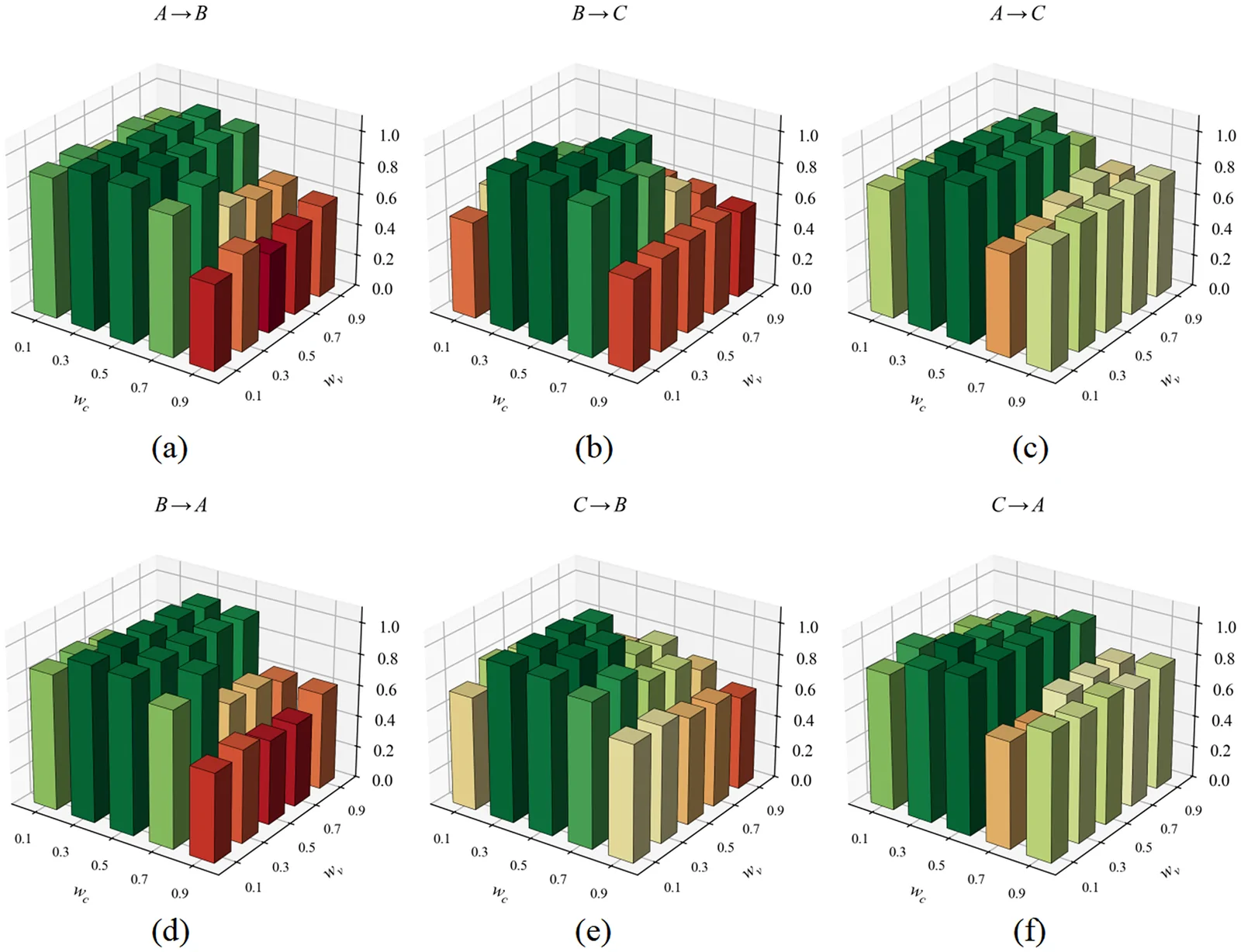
Parameter sensitivity results of $w_{c}$ and $w_{v}$ for XJTU Spurgear dataset. (a) A→B. (b) B→C. (c) A→C. (d) B→A. (e) C→B. (f) C→A.

We conduct an exhaustive grid search over $w_{c}\in \{0.1,0.3,0.5,0.7,0.9\}$ and $w_{v}\in \{0.1,0.3,0.5,0.7,0.9\}$, covering 25 parameter combinations on six cross-sensor transfer tasks (A→B, B→C, A→C, B→A, C→B, C→A) for both the CWRU bearing fault dataset and XJTU Spurgear gear fault dataset. Figs 9 and 10 present the target-domain test accuracy bar charts for each cross-domain transfer task.

**Sensitivity to** $w_{c}$: A pronounced bell-shaped accuracy trend against $w_{c}$ is observed for both datasets. When $w_{c}=0.1$ (too small), the classification loss $L_{c}$ is assigned insufficient weight; the model prioritizes domain distribution alignment excessively and fails to learn discriminative source-domain fault features, resulting in weak classifier fitting and significant accuracy degradation across nearly all transfer tasks on both datasets. In contrast, overly large $w_{c}$ ($w_{c}\geq 0.7$) drastically suppresses the domain adaptation loss $L_{d}$, degenerating the model into a vanilla source-trained classifier that loses cross-domain generalization ability. For the CWRU dataset, high-$w_{c}$ regions are dominated by dark-red low-accuracy zones (accuracy fixed at 0.20 for many grid points), with target-domain accuracy falling to 0.20–0.35; the XJTU dataset also shows a steady accuracy decline at $w_{c}\geq 0.7$. The optimal performance is consistently observed at $w_{c}\in [0.3,0.5]$, where classification supervision and domain alignment loss achieve a favorable trade-off: most grid points of CWRU tasks reach saturated accuracy close to 1.0 in this interval, while most tasks of XJTU Spurgear maintain accuracy above 0.95 and peak above 0.99.

**Sensitivity to** $w_{v}$: Model accuracy exhibits far weaker sensitivity to $w_{v}$ compared with $w_{c}$. Within the optimal $w_{c}\in [0.3,0.5]$ interval, $w_{v}\in [0.1,0.5]$ sustains stable high accuracy for both datasets, verifying effective cooperation between the VDR and MMSD sub-loss terms. Nevertheless, accuracy deteriorates markedly when $w_{v}$ rises to 0.9. For the CWRU dataset under $w_{c}=0.3,w_{v}=0.9$, multiple tasks such as A→C and C→A suffer sharp accuracy collapse to 0.20. An excessively large weight for the VDR sub-loss severely disperses the target-domain feature distribution and breaks intra-class feature compactness for fault samples.

**Cross-dataset consistency:** We calculate task-averaged accuracy for each discrete $w_{c}$ level (sorted as $w_{c}=0.1,0.3,0.5,0.7,0.9$): CWRU = [0.601, 0.970, 0.962, 0.425, 0.305], XJTU Spurgear = [0.765, 0.972, 0.975, 0.815, 0.558]. The two datasets share optimal parameter regions: $w_{c}\in [0.3,0.5]$ paired with $w_{v}\in [0.1,0.5]$. Although the two fault datasets differ in fault mode, vibration signal characteristics, and total sample size, they share similar variation trends of parameter sensitivity.

### 3.7 MMD-based unsupervised hyperparameter selection

The above sensitivity analysis demonstrates that JVM has a well-defined optimal parameter region ($w_{c}\in [0.3,0.5]$, $w_{v}\in [0.1,0.5]$). However, this conclusion relies on target-domain test labels, which are unavailable in practical deployment. To enable hyperparameter selection without target-domain labels, we adopt the Maximum Mean Discrepancy (MMD) as an unsupervised selection criterion. Although MMD was originally a standard distribution discrepancy measure in domain adaptation, it has found growing applications in unsupervised model and hyperparameter selection in recent years [65]. Yang et al. [66] systematically compared MMD and Proxy A-distance for UDA model evaluation, reporting a moderate correlation between MMD and target-domain accuracy.

**MMD criterion definition:** For each grid point $\left(w_{c},w_{v}\right)$ and each transfer task, we compute the distribution distance between the source and target domains in the RKHS using the softmax outputs saved at the final training epoch:


$$\begin{array}{c} {\hat{MMD}}^{2}(S,T)=\frac{1}{n^{2}}\sum_{i,i^{\prime }}k(s_{i},s_{i^{\prime }})+\frac{1}{m^{2}}\sum_{j,j^{\prime }}k(t_{j},t_{j^{\prime }})-\frac{2}{nm}\sum_{i,j}k(s_{i},t_{j}) \end{array}$$ (18)

where $S=\{s_{i}{\}}_{i=1}^{n}$ and $T=\{t_{j}{\}}_{j=1}^{m}$ denote the source-domain and target-domain softmax outputs, respectively, k(·,·) is a Gaussian RBF kernel. A smaller MMD value indicates a closer match between the source and target distributions, i.e., better domain alignment.

**Fig 11:**
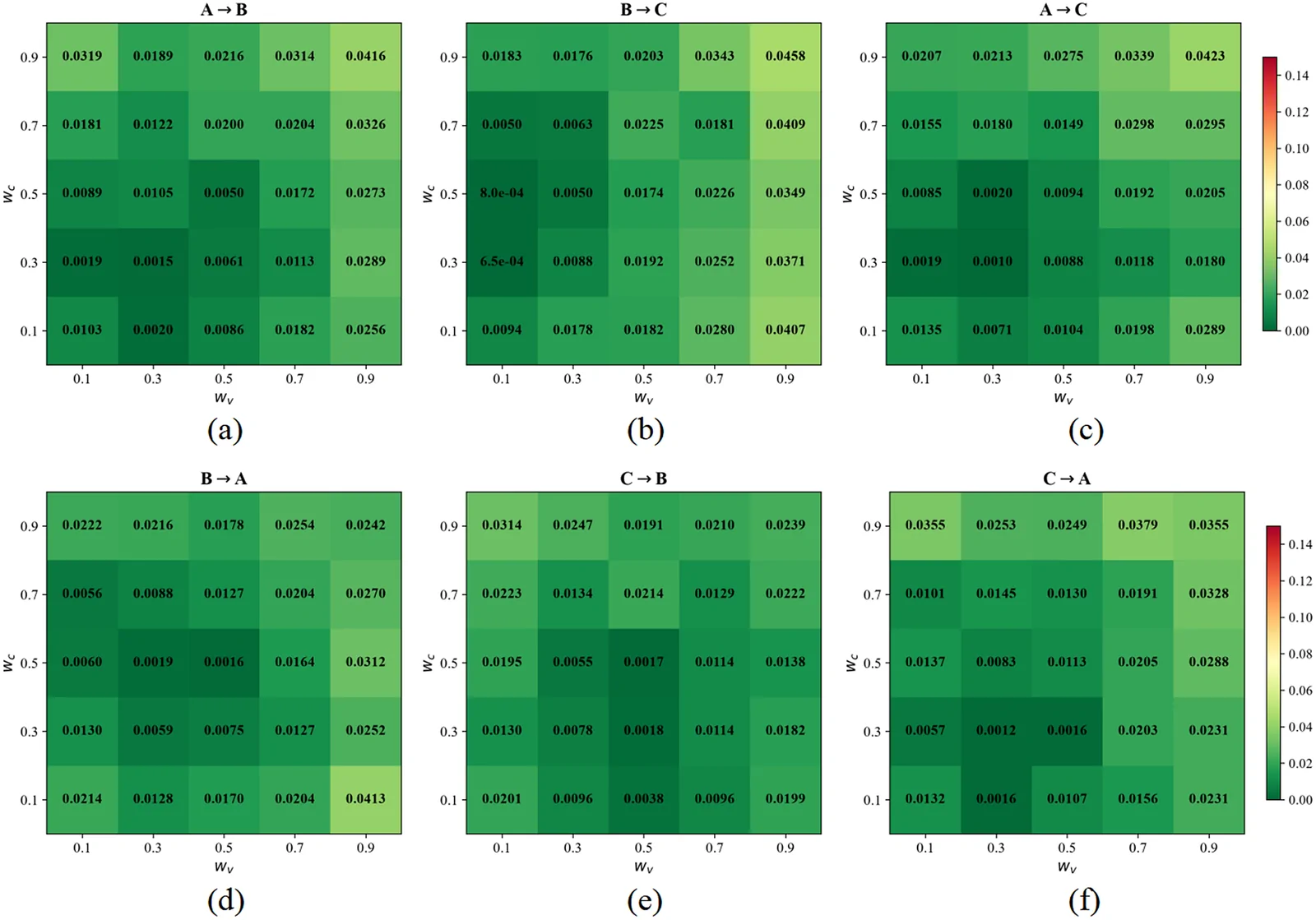
MMD heatmap for CWRU dataset. (a) A→B. (b) B→C. (c) A→C. (d) B→A. (e) C→B. (f) C→A.

**Fig 12:**
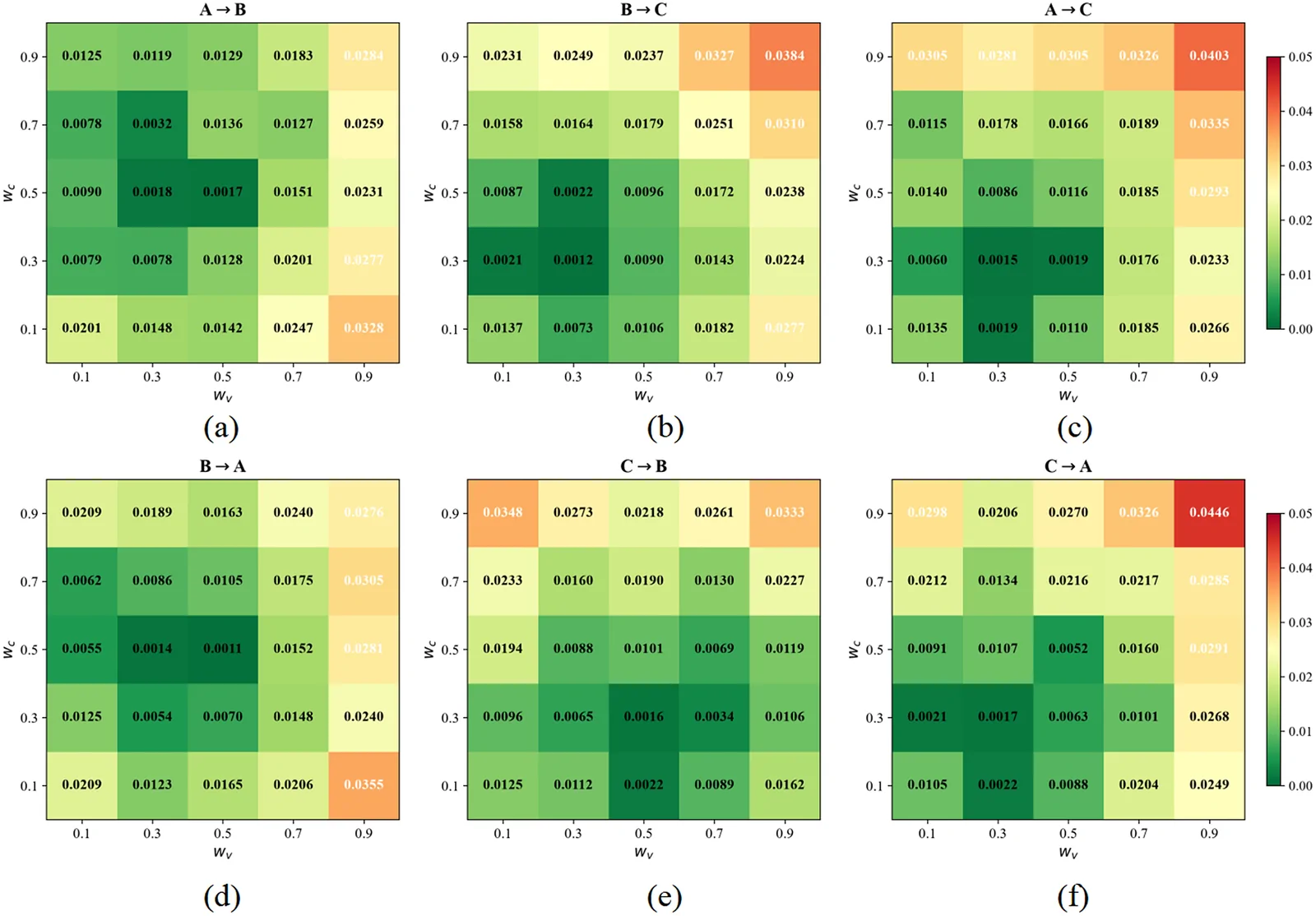
MMD heatmap for XJTU Spurgear dataset. (a) A→B. (b) B→C. (c) A→C. (d) B→A. (e) C→B. (f) C→A.

**MMD heatmap analysis:** Figs 11 and 12 illustrate the per-task MMD heatmaps, which exhibit a pronounced spatial consistency with the target-domain accuracy heatmaps. Three typical regions can be identified as follows:

(i) *Optimal alignment region.* The minimum MMD values (dark green regions) are consistently located in $w_{c}\in [0.3,0.5]$ and $w_{v}\in [0.1,0.5]$, which largely overlap with the highest-accuracy region in the target domain. Within this region, the source and target softmax distributions are well aligned on both datasets, with all MMD values below 0.002 and the minimum dropping to the 10^–4^ magnitude on certain tasks, indicating a near-ideal domain matching state. It is worth noting that although target-domain accuracy enters a saturation plateau in this interval (with marginal improvements as alignment further improves), MMD remains sensitive to fine-grained differences in distribution matching.
(ii) *Pathological alignment region.* When $w_{c}=0.1$, the weight of the classification loss is excessively low, leading to undertrained classifiers and prediction degeneracy (i.e., class collapse) on the target domain. The collapsed target distribution deviates substantially from the multi-class source distribution, which in turn increases the MMD values. This region also corresponds to low accuracy in the accuracy heatmaps, verifying that MMD can correctly distinguish pathological alignment caused by classifier degradation from genuine domain adaptation.
(iii) *Under-alignment region.* When $w_{c}\geq 0.7$, the domain adaptation loss is assigned insufficient weight, such that the feature distributions of the two domains evolve independently without effective alignment. Accordingly, MMD increases markedly (reaching approximately 0.04–0.05 on the CWRU dataset). This region exactly corresponds to the low-accuracy area in the accuracy heatmaps, confirming the sensitivity of MMD to insufficient domain alignment.

**Hyperparameter selection analysis:** Table 5 compares the hyperparameter selection results between the ground-truth optimal (label-guided) and MMD-based unsupervised criteria across all transfer tasks. On the CWRU dataset, 5 out of 6 tasks achieve zero accuracy gap. Even when the selected parameter coordinates differ, MMD still falls within the accuracy saturation plateau and yields the optimal accuracy of 1.0000. Only the task B→C has a minor gap of 0.0234. On the XJTU Spurgear dataset, 3 out of 6 tasks achieve an exact match with zero accuracy gap. Most mismatched cases have gaps below 0.02, and the maximum gap of 0.0356 appears on task A→B. All MMD-selected parameters stay within the effective alignment region. The results validate that MMD can effectively locate the optimal parameter region without target-domain labels, and the corresponding accuracy loss is acceptable in practical deployment.

**Table 5:** Comparison of ground-truth optimal and MMD-based hyperparameter selection.

Dataset	Transfer Task	Ground-truth optimal		MMD-selected		Acc. Gap
		$\left(w_{c},w_{v}\right)$	Accuracy	$\left(w_{c},w_{v}\right)$	Accuracy	
CWRU	A→B	(0.3, 0.3)	1.0000	(0.3, 0.3)	1.0000	0.0000
	B→C	(0.3, 0.3)	1.0000	(0.3, 0.1)	0.9766	0.0234
	A→C	(0.3, 0.3)	1.0000	(0.3, 0.3)	1.0000	0.0000
	B→A	(0.3, 0.3)	1.0000	(0.5, 0.5)	1.0000	0.0000
	C→B	(0.3, 0.3)	1.0000	(0.5, 0.5)	1.0000	0.0000
	C→A	(0.3, 0.3)	1.0000	(0.3, 0.3)	1.0000	0.0000
XJTU Spurgear	A→B	(0.5, 0.3)	0.9892	(0.5, 0.5)	0.9610	0.0356
	B→C	(0.5, 0.3)	0.9976	(0.3, 0.3)	0.9910	0.0066
	A→C	(0.3, 0.3)	0.9985	(0.3, 0.3)	0.9985	0.0000
	B→A	(0.3, 0.3)	0.9966	(0.5, 0.5)	0.9785	0.0181
	C→B	(0.5, 0.3)	0.9980	(0.3, 0.5)	0.9873	0.0107
	C→A	(0.3, 0.3)	0.9985	(0.3, 0.5)	0.9847	0.0138

### 3.8 Online deployment analysis

The proposed 1D Swin Transformer backbone adopts a three-stage hierarchical architecture with depths [2, 4, 2] and PatchMerging between stages, where the feature dimension doubles (128→256→512) and the sequence length halves (3072→1536→768) at each stage transition. The total parameter count is 10.20 M and the computational cost per inference is 11.26 GFLOPs. As shown in Table 6, the FLOPs are distributed across three main components: the attention modules (including QKV projection, score computation, value aggregation, and output projection) account for 3.70 G (32.9%), the MLP modules for 7.25 G (64.4%), and PatchMerging together with the input/output projections for 0.31 G (2.7%).

**Table 6:** Computational profile of the proposed 1D Swin Transformer.

Metric	Value
Architecture	3-stage [2,4,2] + PatchMerging
Parameters	10.20 M
Total FLOPs	11.26 G
Attention (QKV+attn+proj)	3.70 G (32.9%)
MLP	7.25 G (64.4%)
PatchMerging + other	0.31 G (2.7%)
FP32 Latency^†^	3.80 ms
FP32 Memory^†^	71.5 MB
Throughput (FP32)	~260 samples/s
^†^Measured on NVIDIA RTX 4070 SUPER, batch size 1, median of 200 runs.	

The attention component accounts for only one-third of the total FLOPs, which is a direct benefit of the windowed attention mechanism. Within the attention module, the QKV and output linear projections dominate the cost with $𝒪(L\cdot C^{2})$ complexity per layer, whereas the core score-value computation scales as 𝒪(L·W·C). By restricting attention to local windows and propagating information across windows through the shifted-window scheme, the proposed model keeps the attention cost bounded while preserving a global receptive field at the feature level.

We further measured the actual inference latency and memory footprint on an NVIDIA RTX 4070 SUPER GPU. The model achieves a single-sample FP32 inference latency of **3.80 ms** with a peak GPU memory of **71.5 MB**. These results confirm that the proposed model is well suited for online fault diagnosis deployment. The 3.80 ms latency falls well below the 10–100 ms real-time threshold commonly adopted for condition monitoring, supporting a throughput of over 260 samples/s. The 71.5 MB memory footprint fits comfortably within embedded GPU budgets. Even on edge accelerators such as the NVIDIA Jetson Orin series, where computational throughput is typically 5–10× lower than that of desktop GPUs, the projected latency remains within 20–40 ms — still suitable for real-time vibration monitoring in industrial scenarios. Moreover, because the attention cost scales linearly with sequence length, the model can be extended to longer input signals or higher sampling rates without the quadratic latency growth that would afflict a vanilla Transformer.

## 4. Conclusions

Deploying multiple sensors at diverse positions on mechanical equipment to construct multi-point monitoring systems has become a prevailing trend in the field of mechanical fault diagnosis. Nevertheless, discrepancies in the distribution of data between different monitoring points generally require the training of an independent diagnostic model for each sensor, which increases the cost of deployment and reduces practical engineering efficiency. To address this issue, this paper proposes an Unsupervised Cross-sensor Transfer Learning network (UCTL). The developed network transfers fault diagnosis knowledge learned from the source monitoring point (source domain) to the target monitoring point (target domain) without requiring any labeled samples from the target domain, offering a cross-sensor domain-adaptive solution for multi-point mechanical fault monitoring systems. The main contributions of this work are summarized as follows:

1. 1D Swin Transformer Backbone: It directly processes raw one-dimensional vibration signals, capturing both local details and global long-range dependencies, thereby avoiding the need to convert signals into two-dimensional time-frequency images.
2. Joint distribution alignment with weighted VDR-MMSD method: It employs a weighted fusion of Maximum Mean Square Discrepancy (MMSD) and Variance Discrepancy Representation (VDR), both extended to their joint distribution alignment counterparts. By introducing independent weight factors, the model can separately control the alignment strength of the mean squared-kernel and variance-based distribution discrepancies, enabling flexible adaptation to diverse cross-sensor scenarios.
3. End-to-end Unsupervised Transfer Learning Network: It integrates the 1D Swin Transformer and the improved domain loss function into a single network. Since no labels for the target domain sensor data are required during training, the proposed network serves as an end-to-end unsupervised transfer learning network that takes vibration signals as input and outputs diagnostic results.

Based on the CWRU (bearing) and XJTU Spurgear (gear) datasets, three sensor locations (denoted as A, B and C) were selected from each dataset to form six cross-sensor transfer tasks for experiments. The proposed UCTL method demonstrates excellent performance, achieving an average accuracy exceeding 99% across all tasks. t-SNE visualizations show that the feature distributions of the source and target domains are well aligned.
